# Numerical investigation of fluid flow behavior in steel cord with lattice Boltzmann methodology: The impacts of microstructure and loading force

**DOI:** 10.1371/journal.pone.0301142

**Published:** 2024-05-08

**Authors:** Chaojie Zhao, Yanxin Jin, Chaobin Fan, Jun Yang, Rui Wang, Yang Cao

**Affiliations:** 1 Sinopec Research Institute of Safety Engineering Co., Ltd., Qingdao, China; 2 State Key Laboratory of Safety and Control for Chemicals, SINOPEC Research Institute of Safety Engineering Co., Ltd., Qingdao, China; 3 Sinopec Xinan Oilfield Service Corporation, Chengdu, China; UNICAMP, University of Campinas, BRAZIL

## Abstract

Steel cord materials were found to have internal porous microstructures and complex fluid flow properties. However, current studies have rarely reported the transport behavior of steel cord materials from a microscopic viewpoint. The computed tomography (CT) scanning technology and lattice Boltzmann method (LBM) were used in this study to reconstruct and compare the real three-dimensional (3D) pore structures and fluid flow in the original and tensile (by loading 800 N force) steel cord samples. The pore-scale LBM results showed that fluid velocities increased as displacement differential pressure increased in both the original and tensile steel cord samples, but with two different critical values of 3.3273 Pa and 2.6122 Pa, respectively. The original steel cord sample had higher maximal and average seepage velocities at the 1/2 sections of 3D construction images than the tensile steel cord sample. These phenomena should be attributed to the fact that when the original steel cord sample was stretched, its porosity decreased, pore radius increased, flow channel connectivity improved, and thus flow velocity increased. Moreover, when the internal porosity of tensile steel cord sample was increased by 1 time, lead the maximum velocity to increase by 1.52 times, and the average velocity was increased by 1.66 times. Furthermore, when the density range was determined to be 0–38, the pore phase showed the best consistency with the segmentation area. Depending on the Zou-He Boundary and Regularized Boundary, the relative error of simulated average velocities was only 0.2602 percent.

## 1. Introduction

With the rapid advancement of the automobile industry, the importance of tire quality in the operation of automobiles has escalated. Mitigating tire failures, extending tire lifespan, and enhancing tire safety have become crucial areas of focus in tire research. Significantly, the molding quality of cord-rubber materials plays a pivotal role in determining overall tire quality [1–3]. The fluid flow behavior within the steel cord material is directly linked to the potential formation of bubbles and cord delamination [4, 5].

Nondestructive testing technology, particularly industrial computed tomography (CT), holds significance in detecting internal defects and precisely measuring internal structures [6–12]. Schock et al. [13] suggested a combination of high-resolution dual-energy X-ray micro-CT with subsequent advanced image processing steps to derive characteristic parameters for describing real porous systems. Liu et al. [14] proposed the reconstruction of a microscopic heat transfer model through CT images to investigate the interaction between coal temperature rise fracturing and coal heat transfer performance, introducing a novel approach for efficient heat injection mining of coal seams. Singh et al. [15] introduced a texture characterization method named Gray-Size Area Matrix (GLSZM), directly applied to the original gray-level micro-CT images capable of reproducing these images with reduced time and high computational efficiency.

Numerous studies have been conducted on seepage mechanics by scholars from diverse countries [16–25]. The lattice Boltzmann method (LBM) has gained considerable traction in resolving flow issues characterized by significant micro-scale effects and intricate channels [26–34]. Guo et al. [35] introduced a lattice Boltzmann model for isothermal incompressible flow within porous media. This model incorporated porosity into the equilibrium distribution function and introduced a force term into the evolution equation, thereby coupling the medium resistance term of porous media with the standard lattice Boltzmann model. Building upon Guo’s model, Gao et al. [36] adjusted the porosity by considering its variability in relation to mineral composition. They developed a characterization-cell lattice Boltzmann model capable of simulating multiple mineral components concurrently. Martin et al. [37] proposed a methodology for simulating multiphase flow in heterogeneous porous media using the lattice Boltzmann method, combining grayscale with a multi-component single equation approach. Saadat et al. [38] restored the Galilean invariance and isotropy of the stress tensor by introducing extended equilibria, expanding the lattice Boltzmann model to handle simulations with higher flow rate values, and enhancing computational efficiency by reducing the required number of time steps. Zhao et al. [39] employed micron CT scanning imaging experiments and the lattice Boltzmann simulation method to qualitatively and quantitatively characterize of the microscopic pore structure of tight sandstone and conduct flow simulations, consequently determining the permeability of tight sandstone samples. The findings bear significant reference value for understanding the microscopic pore structure of tight sandstone.

**Fig 1:**
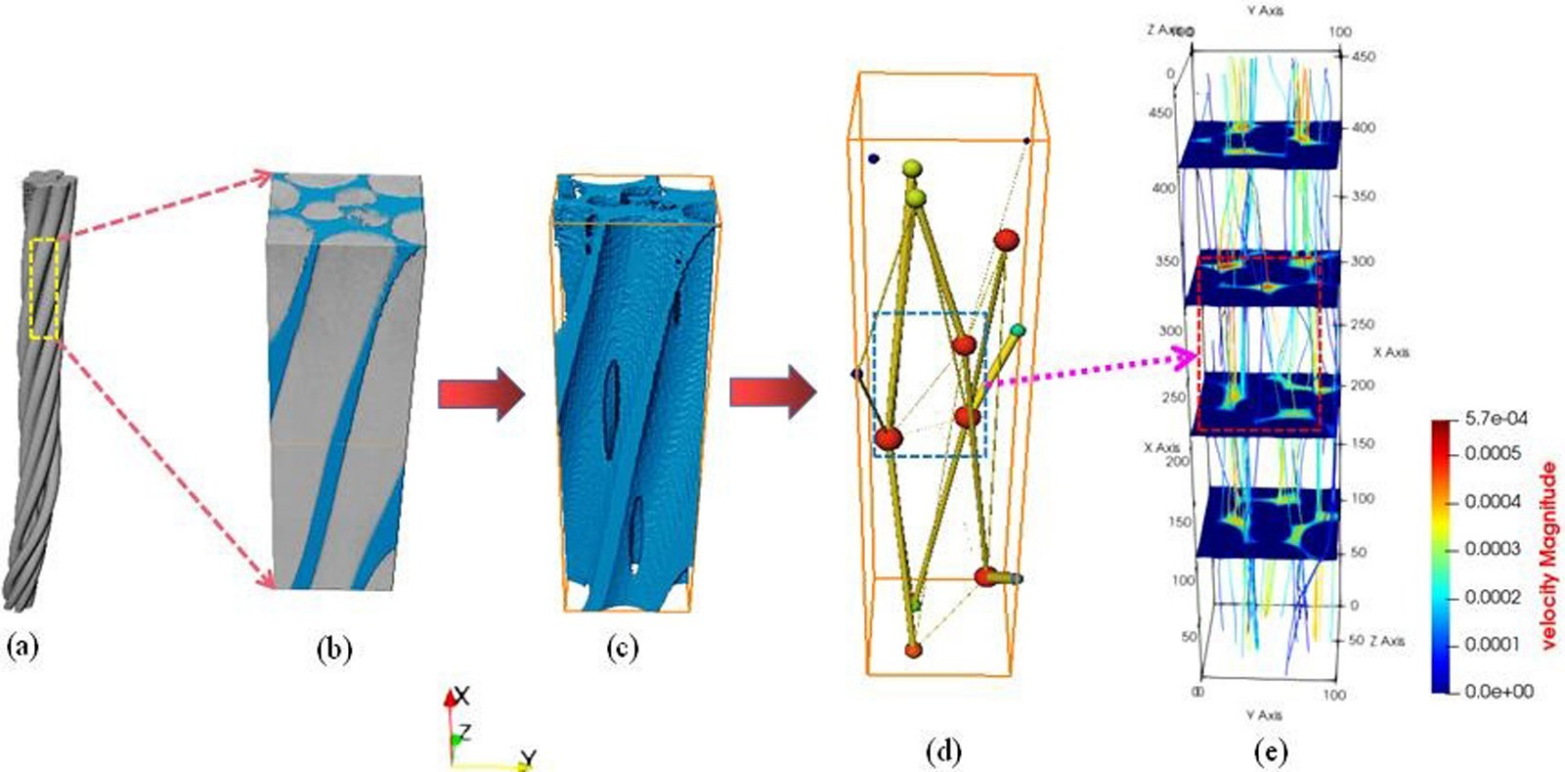
The detailed research thought of this study.

While many scholars have applied CT scanning technology and the LBM approach to analyze permeability in various porous media, research focusing on steel cord materials remains limited. This study utilizes CT scanning technology alongside the LBM simulation method to analyze the genuine three-dimensional (3D) pore structures and fluid behaviors within steel cord materials pre- and post-stretching. Microscopic fluid dynamics in both the original and stretched steel cord materials are visually represented through two-dimensional (2D) velocity slices and 3D flow field distribution diagrams derived from pore-scale and representative elementary volume (REV)-scale LBM analyses. By scaling up, the investigation unveils how pore structure parameters and fluid properties influence flow behaviors within the steel cord porous medium. Offering theoretical insights, this study contributes a microscopic understanding of fluid behavior within steel cord materials, potentially enhancing product quality. For an in-depth understanding of the research methodology, please refer to Fig 1. Fig 1(A) depicts the CT scanning results of the original steel cord sample. Fig 1(B) shows a representative local area taken from the original sample. Fig 1(C) depicts the pore model extracted by threshold segmentation technology, which would be employed as the porous medium for further velocity simulation by LBM. Fig 1(D) depicts the pore network model generated using the maximum sphere algorithm. Fig 1(E) depicts a flow diagram with four stratified slices obtained by LBM simulation.

## 2. Methodology

### 2.1 CT reconstruction

**Fig 2:**
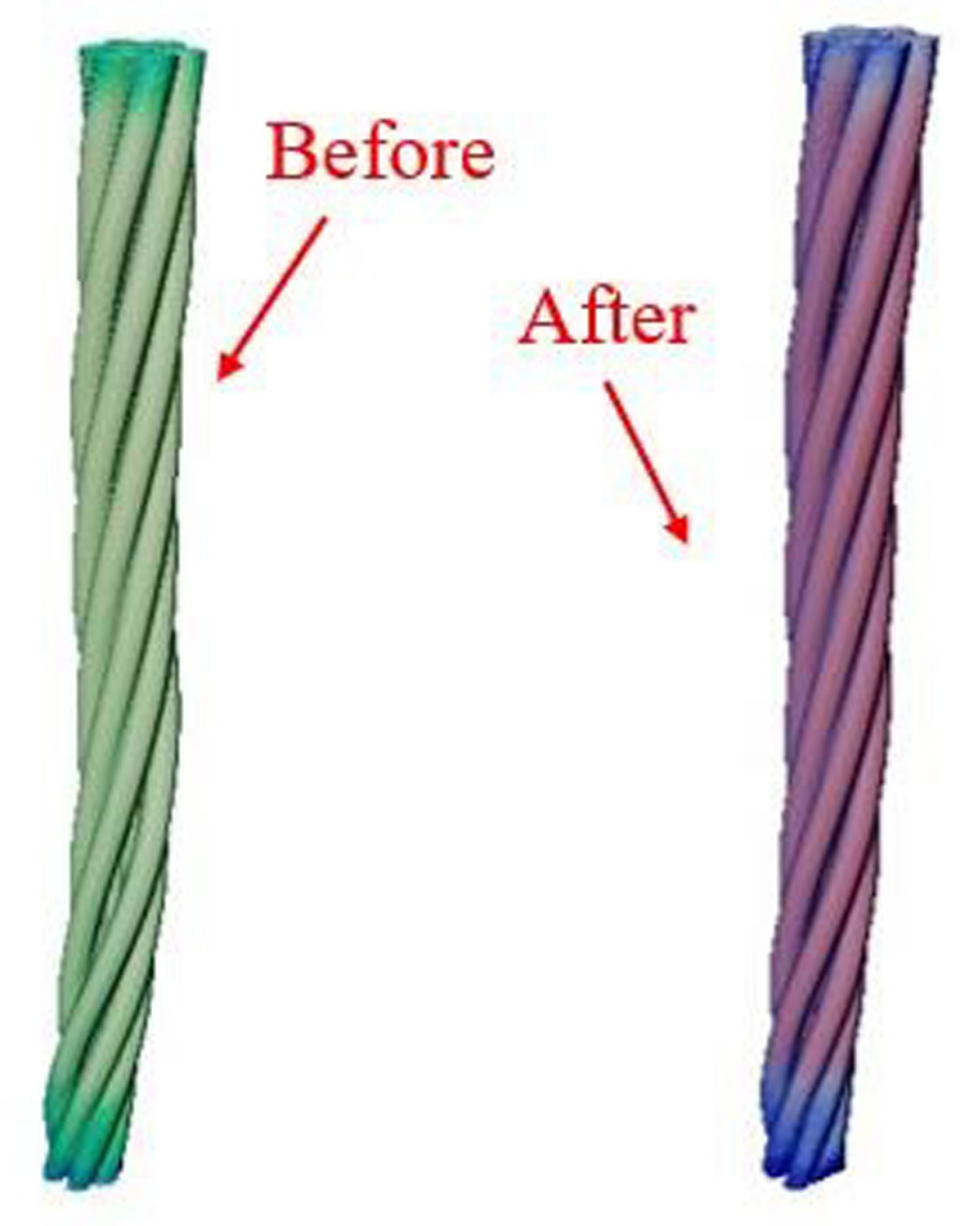
Scan images of the original and stretched samples.

In this study, the microscopic pore structures within steel cord materials were examined using high-resolution 3D nondestructive X-ray microtomography. The research encompassed both the original steel cord sample and those subjected to tension. Applying an 800 N force transformed the original steel cord sample into the tensile steel cord sample. The base of the original steel cord sample remained secured while the tensile steel cord sample exhibited an elongation of 0.155 mm. Fig 2 showcases partial sections obtained from the scanning process, depicting both the original and tensile steel cord samples.

### 2.2 LBM simulation

The LBM is a numerical method used to simulate fluid dynamics. It is based on the Boltzmann equation, but uses discrete velocity space and time-space lattices. In LBM, the velocity space is represented by a discrete set of lattice velocities, usually D2Q9 or D3Q27, and the distribution is represented by a lattice in two—and three-dimensional space, with each lattice velocity being a weight associated with it.

D2Q9 lattice velocity vector (coordinates $c_{i_{x}}$, $c_{i_{y}}$, $c_{i_{z}}$), where *v*_0_ = (0,0,0), *v*_1_ = (1,0,0), *v*_2_ = (0,1,0), and so on; The weight, $w_{0}=\frac{8}{27}$, $w_{1}=w_{2}=w_{3}=w_{4}=w_{5}=w_{6}=\frac{1}{27}$, $w_{7}=w_{8}=\frac{1}{54}$, here $c_{i_{x}}$, $c_{i_{y}}$, $c_{i_{z}}$ is the coordinates of the velocity component, and in both cases the weight is chosen according to preserving the conservation of mass and momentum.

In LBM, the distribution function *f* describes the probability density function at each cell speed. For D2Q9 cells, there are 9 distribution functions on each cell. The general form is *f*_*i*_(*x*, *t*), where *i* is the index of the velocity component, *x* is the spatial position, and *t* is the time.

In the process of simulation, it is necessary to set the initial distribution function, and the common choice is to set the initial distribution function according to the equilibrium distribution function, that is, the system is in equilibrium at the beginning. The general form of the equilibrium distribution function is:

$$f_{i}^{eq}(x,t)=\omega_{i}\rho (1+\frac{v_{i}\cdot u}{c_{s}^{2}}+\frac{{\left(v_{i}\cdot u\right)}^{2}}{2c_{s}^{4}}-\frac{u\cdot u}{2c_{s}^{2}})$$ (1)

Where, *ω_i_* is the weight, *ρ* is the density, *u* is the velocity, *v*_*i*_ is the velocity vector on the lattice, *c*_*s*_ is the speed of sound.

In collision operations, the distribution function is updated according to the collision model. Common collision models include the single relaxation time (BGK) model. The general form of collision operation is:

$$f_{i}(x,t+\Delta t)=f_{i}(x,t)-\frac{1}{\tau }\left[f_{i}(x,t)-f_{i}^{eq}(x,t)\right]$$ (2)

Where, *f*_*i*_(*x*, *t*) is the distribution function on the velocity component, Δ*t* is the time step, *τ* is the relaxation time, is a parameter controlling the collision relaxation rate; $f_{i}^{eq}(x,t)$ is an equilibrium equation, usually calculated from local macroscopic quantities such as density and velocity.

The transport operation simulates the transfer of the distribution function in the flow field by updating the distribution function according to the current distribution function and the velocity field. The general form of the transport operation is:

$$f_{i}(x+v_{i}\cdot \Delta t,t+\Delta t)=f_{i}(x,t)$$ (3)

Indicates that a particle moves to an adjacent lattice point at its velocity within a time step.

The Boltzmann equation describes the evolution of particle distribution function in the physical momentum space, which is commonly written as:

$$\frac{\partial f}{\partial t}+\xi \frac{\partial f}{\partial x}+a\frac{\partial f}{\partial \xi }=\Omega (f)$$ (4)

A simplified BGK model of collision terms is adopted:

$$\Omega =-\frac{f-f^{eq}}{\tau }$$ (5)

Hence, the Boltzmann equation can be reduced to:

$$f(x+e\delta t,e,t+\delta t)-f(x,e,t)=-\frac{1}{\tau }\left[f(x,e,t)-f^{eq}(x,e,t)\right]$$ (6)

The classical D3Q19 phase space model is used in the three-dimensional space, and the discrete velocity set, lattice velocity and weight coefficient are determined:

$$e_{i}=c\left(\begin{array}{ccc} 0 & 1 & \begin{array}{cc} \begin{array}{cc} \begin{array}{cc} -1 & 0 \end{array} & 0 \end{array} & \begin{array}{cccc} 0 & 0 & 1 & \begin{array}{cccc} -1 & -1 & 1 & \begin{array}{cccc} 1 & -1 & -1 & \begin{array}{cccc} 1 & 0 & 0 & \begin{array}{cc} 0 & 0 \end{array} \end{array} \end{array} \end{array} \end{array} \end{array}\\ 0 & 0 & \begin{array}{cccc} 0 & 1 & \begin{array}{cccc} -1 & 0 & 0 & \begin{array}{cccc} 1 & 1 & -1 & \begin{array}{cccc} -1 & 0 & 0 & 0 \end{array} \end{array} \end{array} & \begin{array}{cccc} 0 & 1 & \begin{array}{cc} -1 & -1 \end{array} & 1 \end{array} \end{array}\\ 0 & 0 & \begin{array}{cccc} 0 & 0 & \begin{array}{cccc} 0 & 1 & -1 & \begin{array}{cccc} 0 & 0 & 0 & \begin{array}{cccc} 0 & 1 & 1 & -1 \end{array} \end{array} \end{array} & \begin{array}{cccc} -1 & 1 & \begin{array}{cc} 1 & -1 \end{array} & -1 \end{array} \end{array} \end{array}\right)$$ (7)

$$c=\frac{\delta x}{\delta t}$$ (8)

$$w_{i}=\left\{\begin{array}{l} 1/3,e_{i}^{2}=0\\ 1/18,e_{i}^{2}=c^{2}\\ 1/36,e_{i}^{2}=2c^{2} \end{array}\right\}$$ (9)

The macroscopic fluid density, velocity, pressure and viscosity coefficient are derived from the equations:

$$\left\{\begin{array}{l} \rho =\sum_{i}f_{i}\\ u=\sum_{i}\frac{f_{i}e_{i}}{\rho }\\ p=c_{s}^{2}\rho v=\frac{c^{2}}{3}(\tau -0.5)\delta t \end{array}\right.$$ (10)

### 2.3 Boundary condition

**Fig 3:**
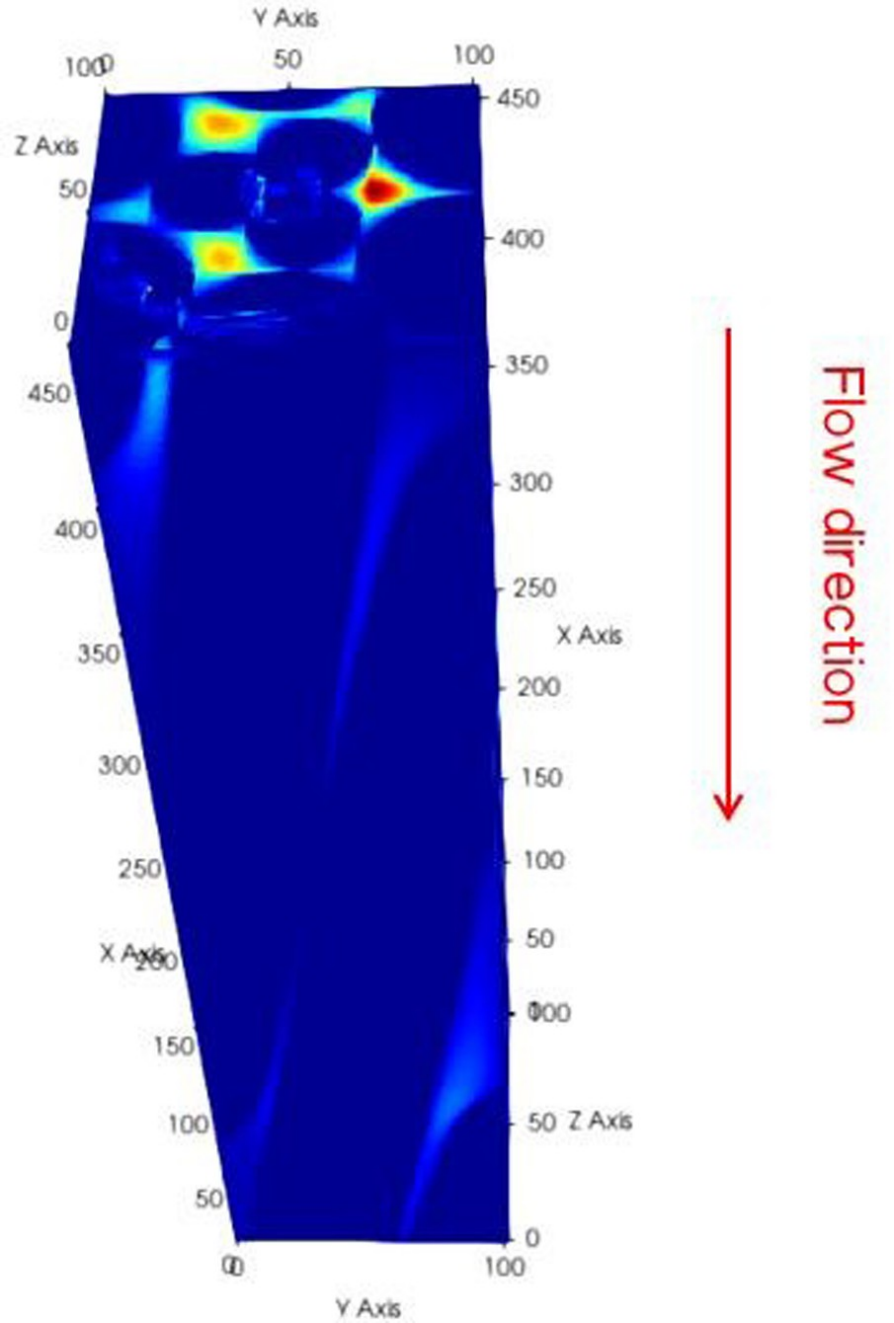
Diagram of water flow in a sample of steel cord.

During the numerical iteration process, an assumption was made that water flowed driven by pressure from the top to the bottom of the steel cord materials. Fig 3 illustrates the steel cord material in blue and highlights the water seepage area in color. To simulate this, the Zou-He Boundary and Regularized Boundary were employed, while standard rebound conditions were set up on the left and right sides.

### 2.4 Methodology verification

In this section, the flow mechanism within a 10 mm diameter circular tube was simulated using the LBM. The displacement differential pressures were set at 0.00001, 0.00002, and 0.00003 within the lattice cell unit.

Notably, the x-direction velocity within a circular tube can be calculated using the Eq (11):

$$u_{x}=\frac{\nabla P_{x}}{\mu }\left(\frac{D^{2}}{16}-\frac{r^{2}}{4}\right)$$ (11)

**Fig 4:**
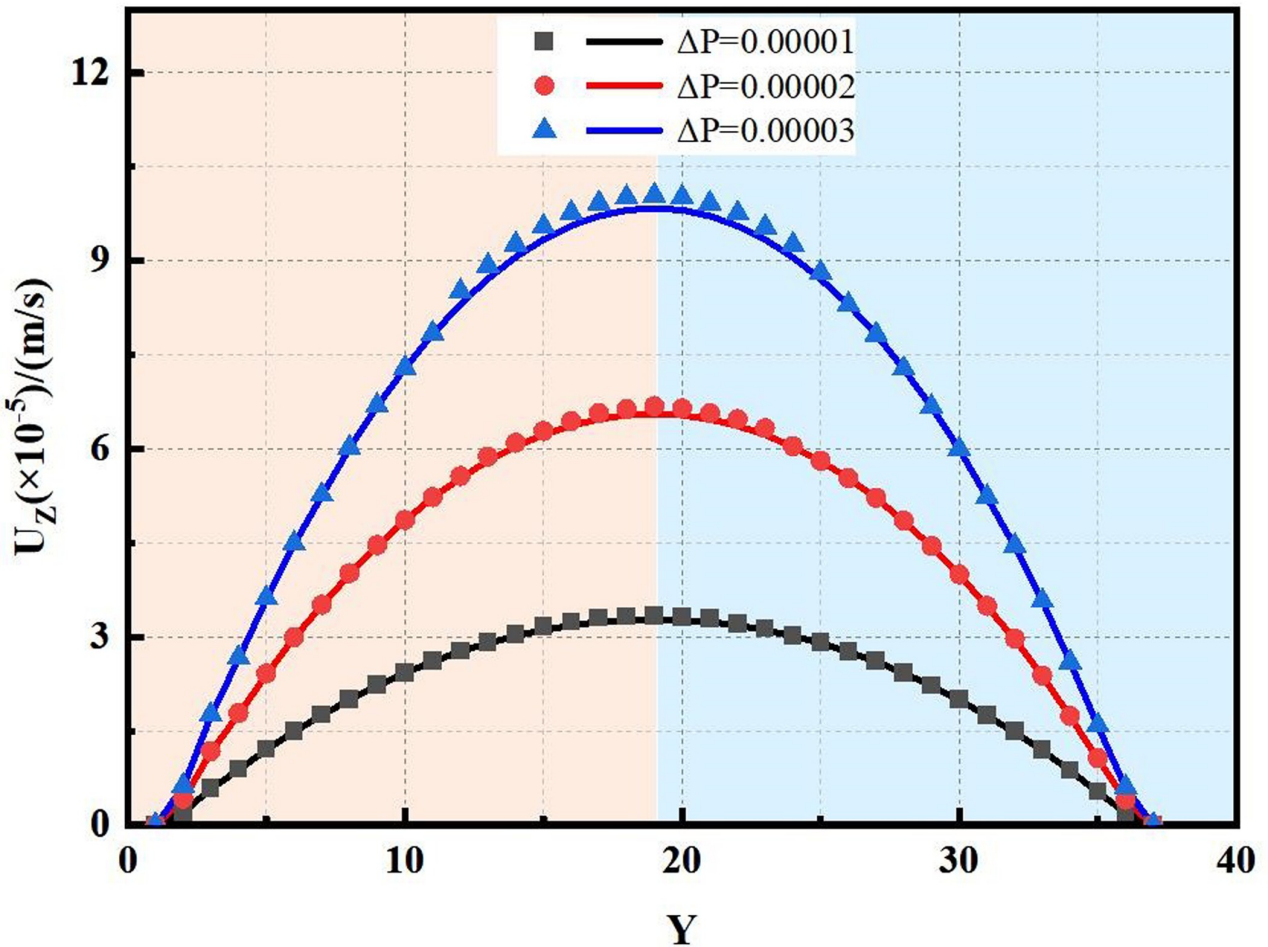
Comparison results by the LBM and analytical method.

According to Fig 4, the flow velocities determined by the LBM was in good agreement with those derived from the analytical solution (Eq (11)). The maximum errors observed at three distinct displacement differential pressures were all below 10%.

## 3. Results and discussions

### 3.1 Pore-scale LBM results

#### 3.1.1 Quantitative characterization of pore structure

**Fig 5:**
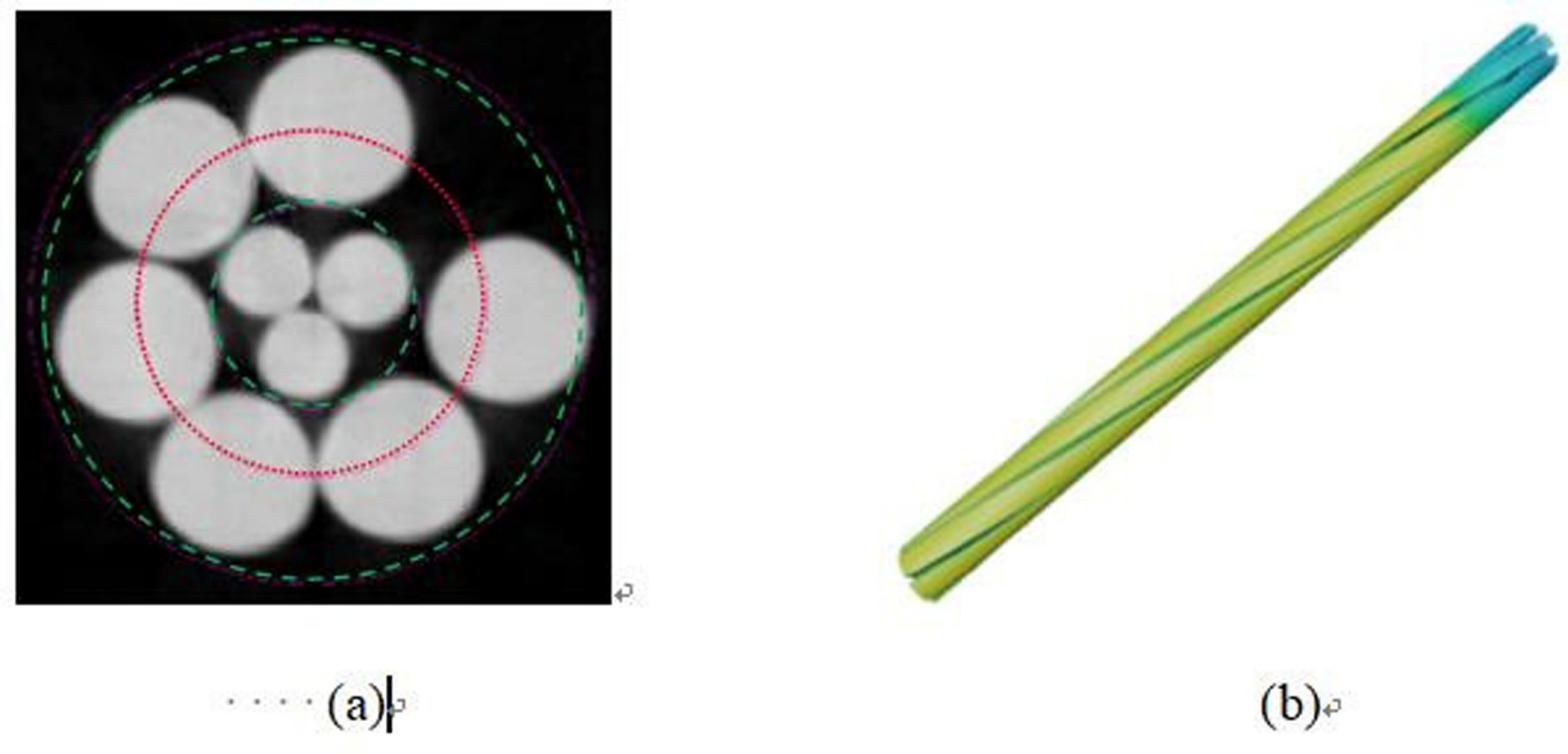
Images of the original steel cord sample.

The CT scanning process generated numerous 2D images representing the steel cord material. To ensure an accurate representation of the actual pore structures within the steel cord material, circular slices from the middle of all images were selected for subsequent 3D reconstruction. The designated research area was determined as a cylindrical frame with a radius of 363.85 μm and a height of 11373.9 μm. The inner circle had a diameter of 220.63 μm, while the outer circle’s diameter was 569.84 μm. Fig 5(A) displays the chosen circular area extracted from these slices, while Fig 5(B) illustrates the resulting 3D reconstruction of the steel cord sample based on the selected circular area.

The pore structures were isolated from the 3D entity through threshold segmentation. This process involved creating a pore model by subtracting a solid cylinder from the entity obtained via threshold segmentation. Employing the volume fraction command, the porosity of the original steel cord sample was calculated to be 26.94 percent. The specific pore parameters were itemized and presented in Table 1.

**Table 1:** Pore parameters of the original steel cord sample.

Parameters	Pore radius/μm	Pore volume/μm^3^	Pore surface area/μm^2^
Maximum	60.62	43645.70	12260.40
Average	6.36	3320.58	1230.14
Minimum	0.53	609.41	2.56

**Fig 6:**
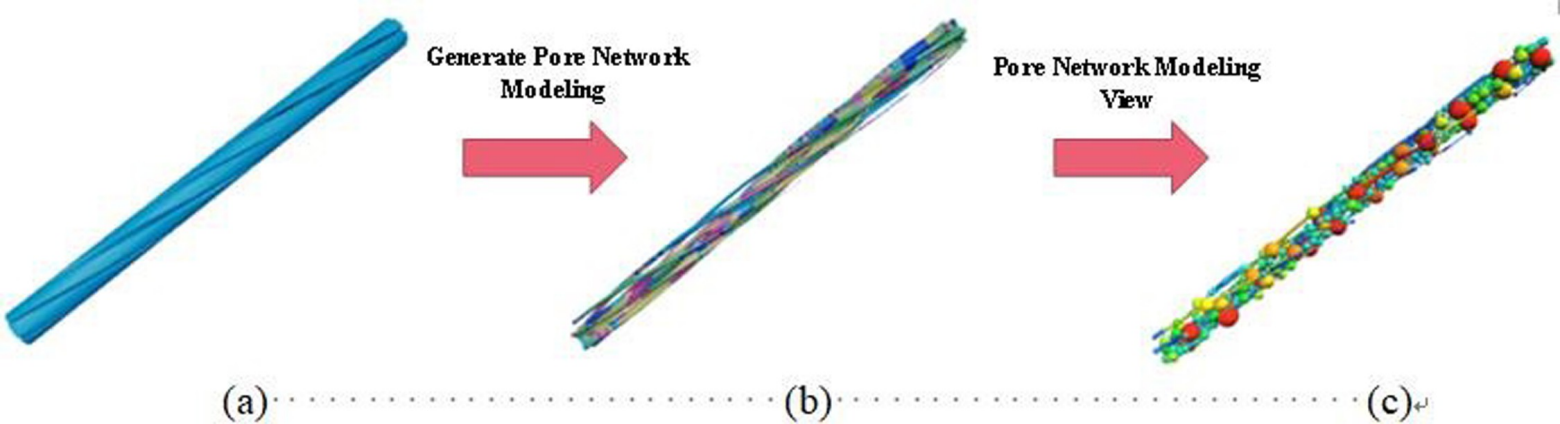
The pore network model of original steel cord sample.

**Fig 7:**
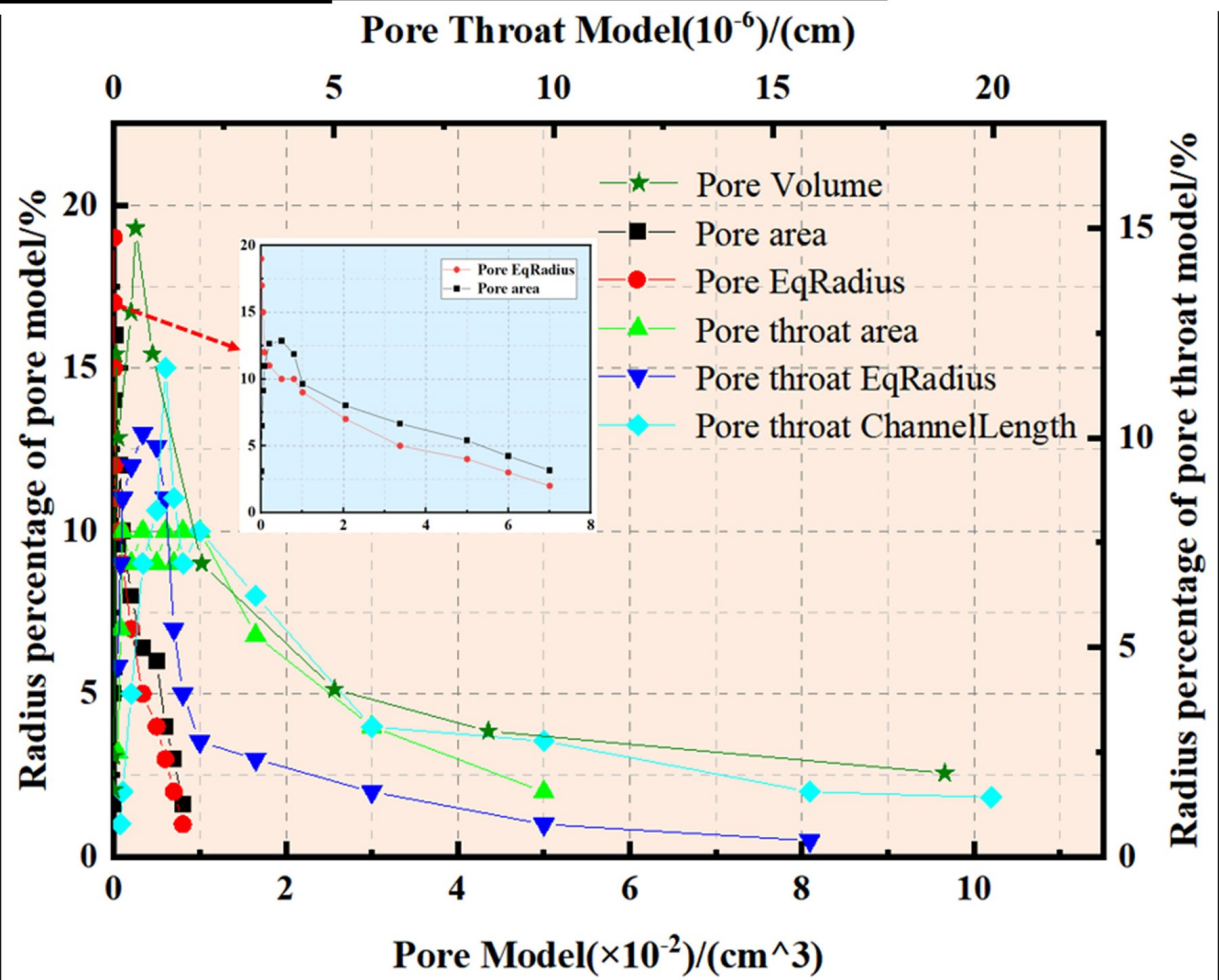
Results of pore-throat in original sample.

To elucidate the internal pore structure characteristics of the original steel cord sample, establishing a comprehensive pore network model becomes imperative. Fig 6 illustrates the pore network model, employing spheres to denote internal pores and cylinders to represent internal throats. Notably, the cylinder thickness is proportionate to the throat radius, reflecting the sphere’s size. Concurrently, Fig 7 quantifies the characteristic parameters of pore and throat structures observed within the original steel cord sample.

The methodology employed for the 3D reconstruction and pore network modeling of the tensile steel cord sample mirrored the previously outlined approach. The research area encompassed a cylindrical frame measuring 363.85 μm in radius and 11373.9 μm in high. Within this space, the inner circle displayed a diameter of 220.63 μm, while the outer circle diameter expanded to 569.84 μm. The porosity analysis of the tensile steel cord sample revealed a calculated value of 17.69%. Detailed pore parameters specific to the tensile steel cord samples are presented in Table 2.

**Table 2:** Pore parameters of the tensile steel cord sample.

Parameters	Pore radius/μm	Pore volume/μm^3^	Pore surface area/μm^2^
Maximum	129.94	10724.17	4963.32
Average	7.60	1413.58	582.05
Minimum	3.58	191.50	99.81

**Fig 8:**
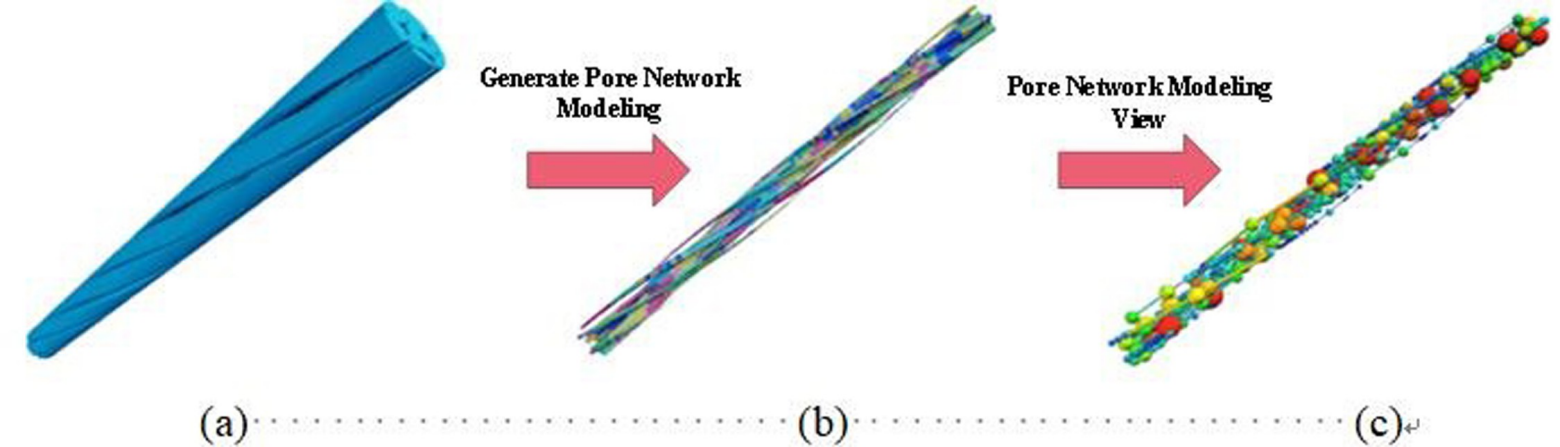
Pore network model of tensile sample.

**Fig 9:**
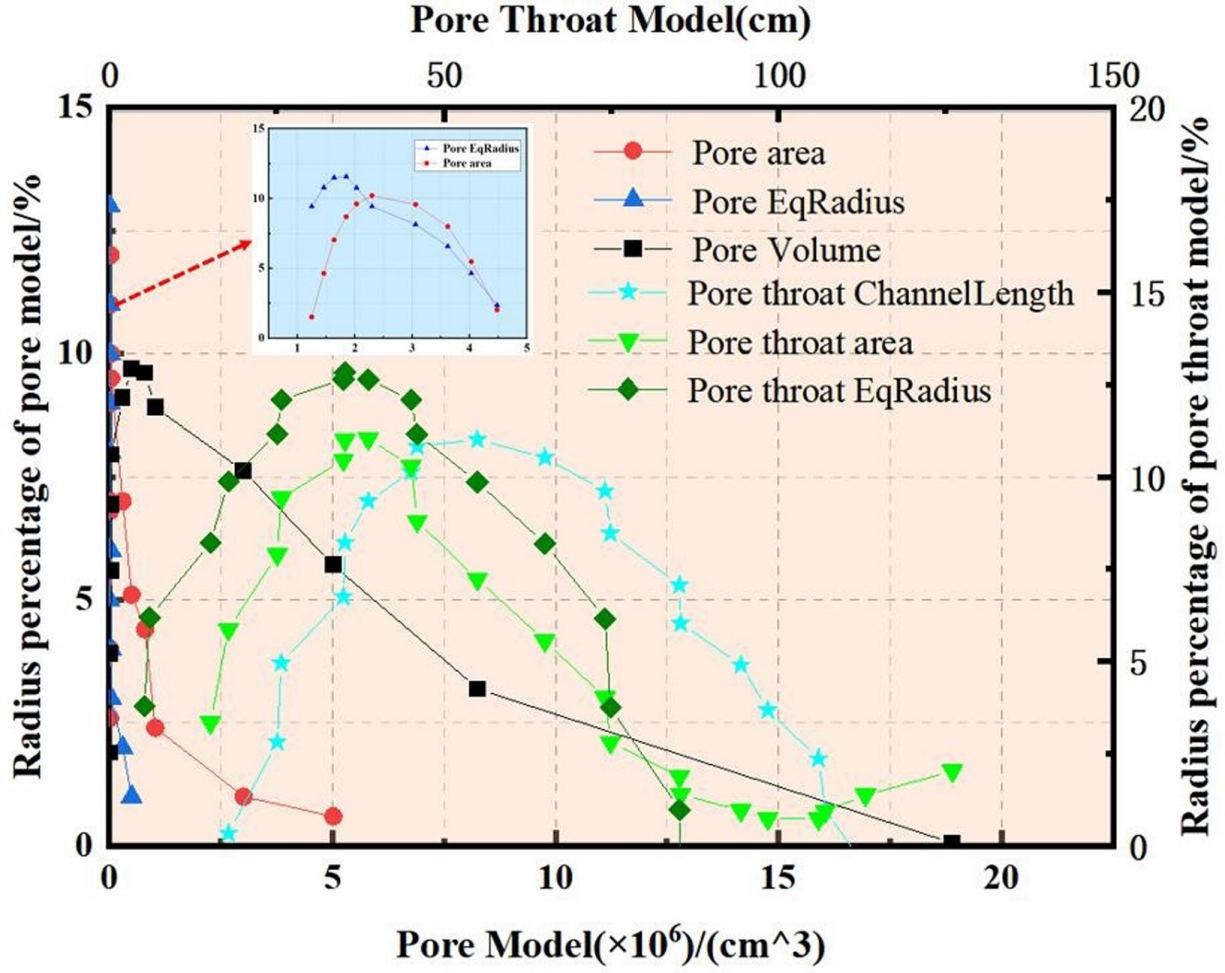
Results of pore-throat in tensile sample.

Fig 8 presents the pore network model corresponding to the tensile steel cord sample, while Fig 9 quantitatively analyzes the characteristic parameters of pore and throat structures within this sample. A comparative examination between Figs 7 and 9 reveals a consistent distribution trend in the pore structures of the steel cord materials, indicating similarity before and after loading.

#### 3.1.2 Fluid flow characteristics

We employed the LBM approach to explore the effects of displacement differential pressure and boundary condition on water flow behaviors within the original steel cord material, building upon the earlier 3D reconstruction of the material. In the LBM simulation, both Zou-He Boundary and Regularized Boundary methods were utilized. 455 sections, each comprising 102×102 pixels from the entire CT scan of the steel cord sample, were integrated into the simulation process. This process generated both 2D flow slice distribution diagrams and 3D flow field distribution diagrams. The observed phenomenon can be attributed to the enhanced homogeneity of pore structures within the original steel cord sample, aligning perfectly with the actual seepage mechanism—Darcy’s law.

**Fig 10:**
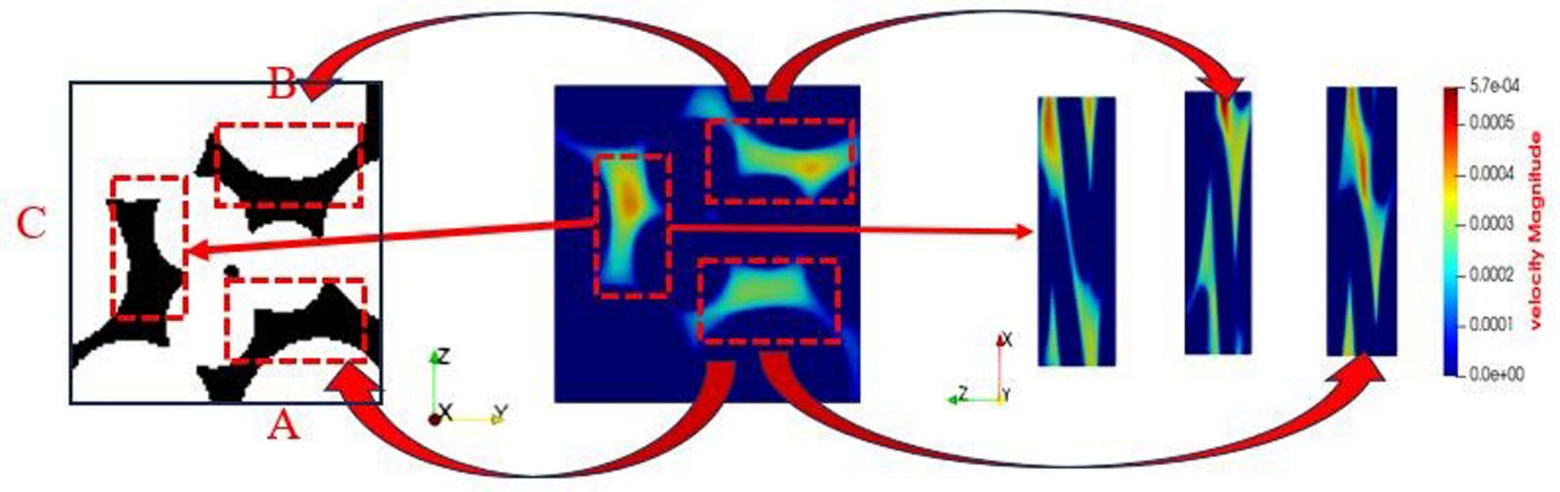
Flow channel structure of original sample.

**Fig 11:**
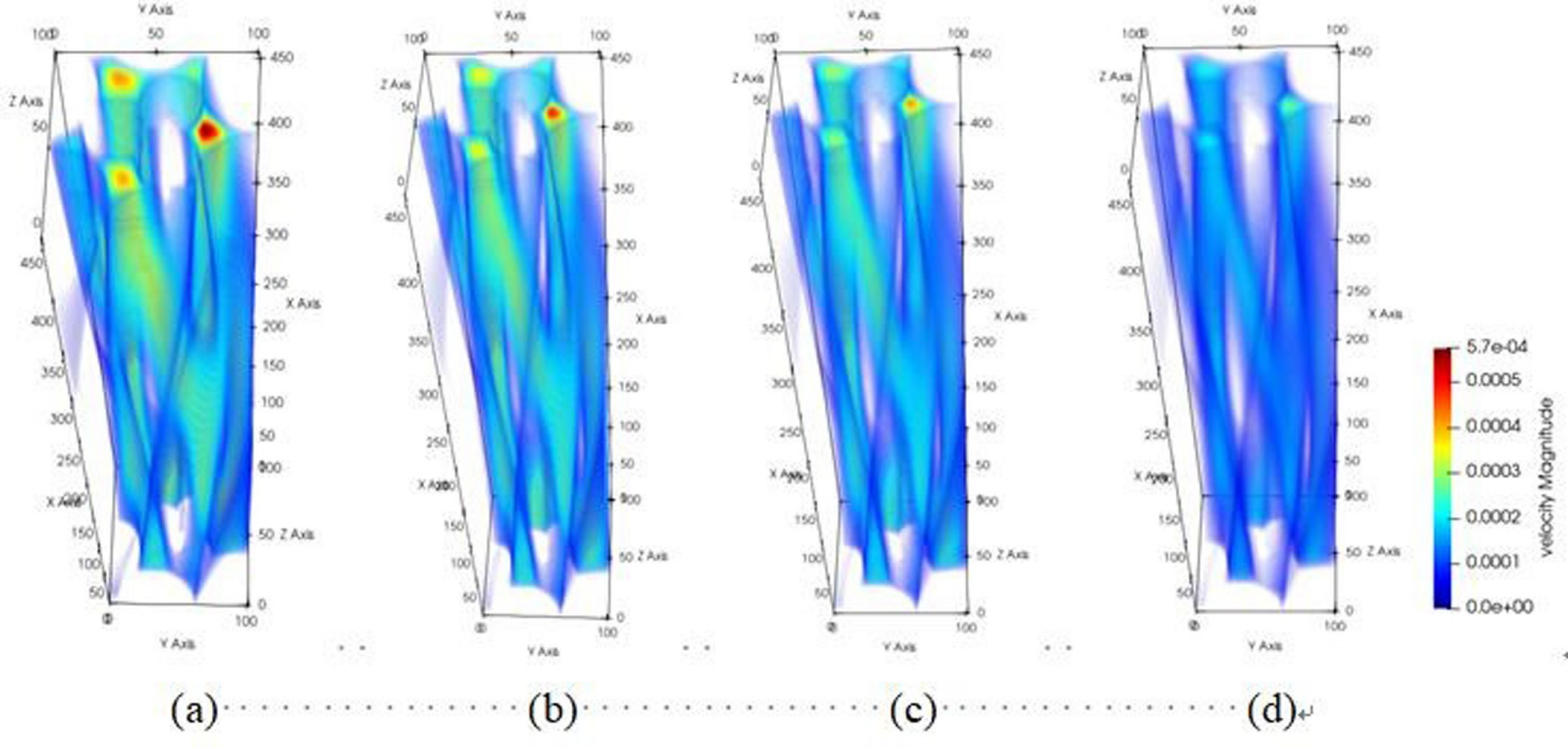
Results of 3D flow field of original sample.

**Fig 12:**
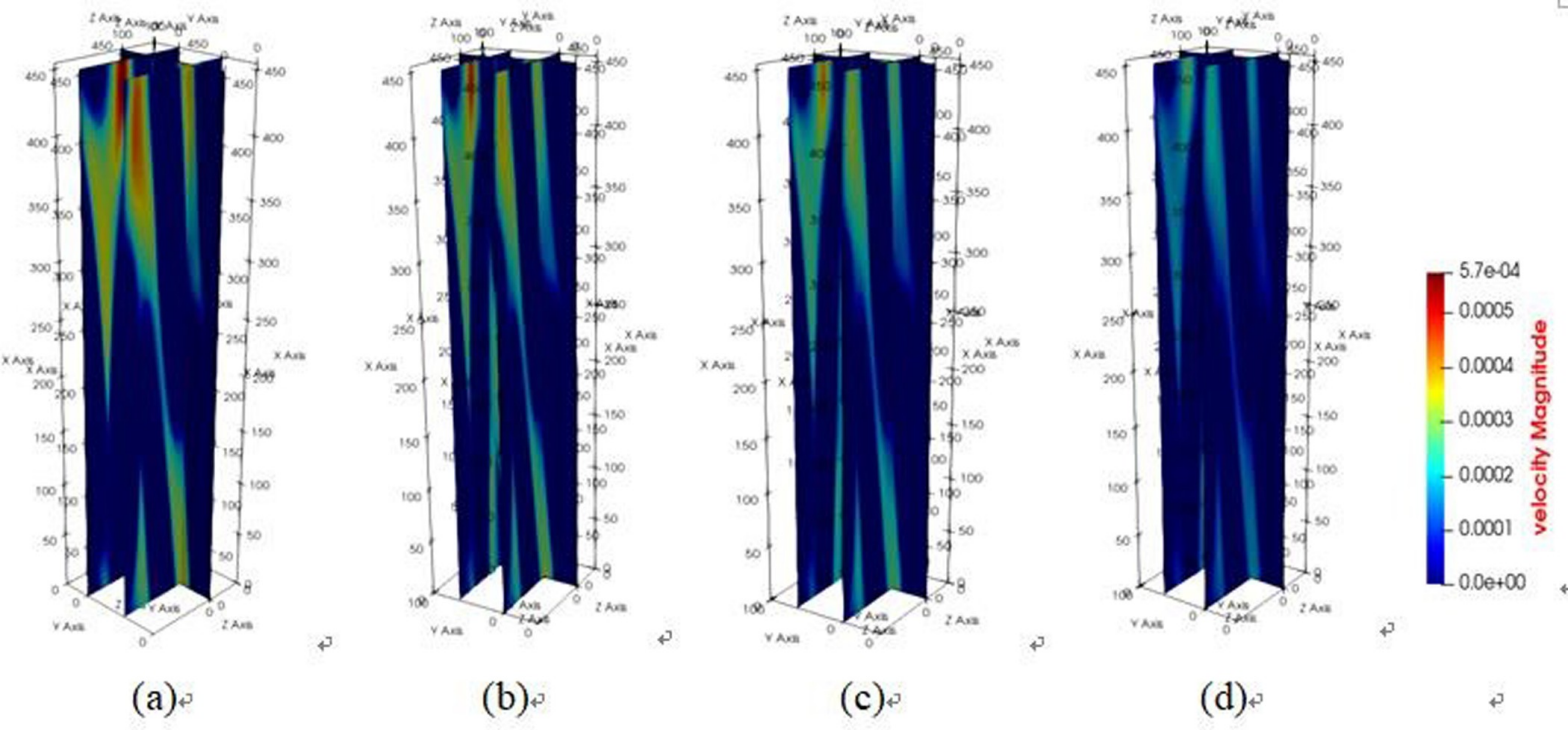
Results of 2D flow slice of original sample.

**Fig 13:**
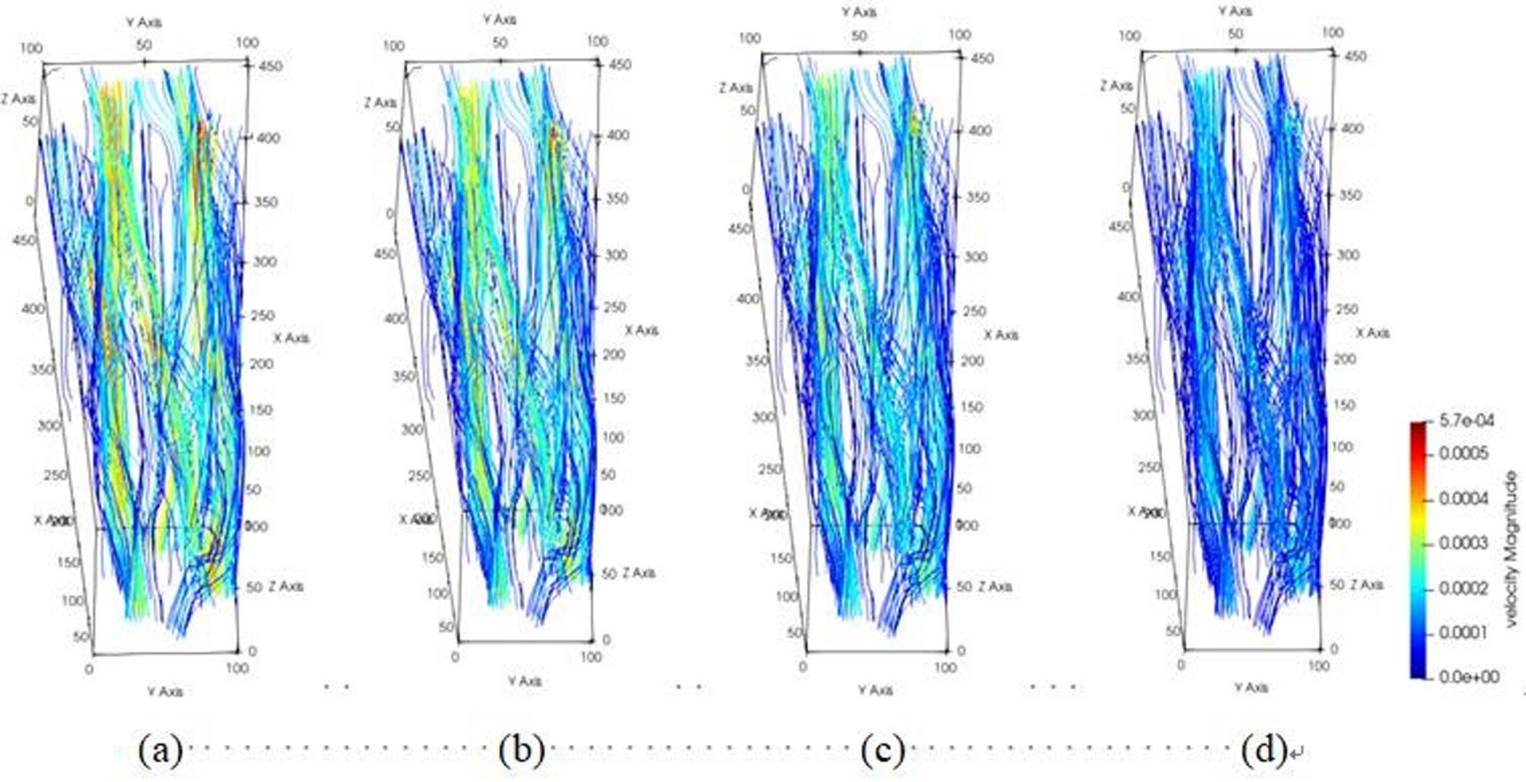
Results of 3D streamline of original sample.

**Fig 14:**
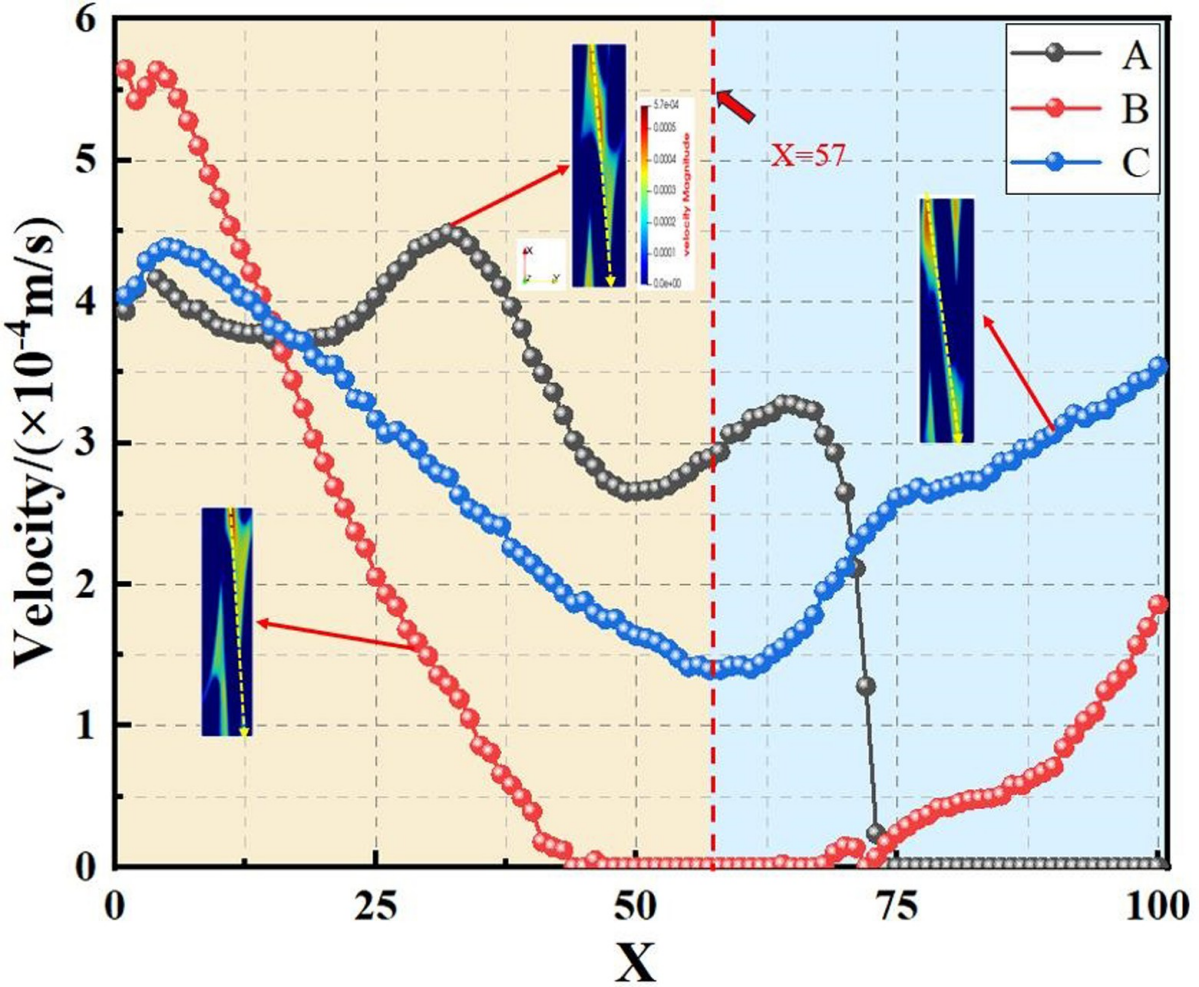
Results of three-channel curve in original sample.

The pore channel structure distribution within the original steel cord material predominantly comprises three segments: flow channels A, B, and C, as represented in Fig 10. Fig 11 illustrates the 3D flow field distributions under four distinct displacement differential pressures, providing a detailed view of the velocity distribution near the solid wall. Additionally, the yz-directional velocity field extracted from the original steel cord sample using 2D slices exposes the material flow characteristics. Fig 12 demonstrates the 2D flow slices with four displacement pressure differences, and Fig 13 shows the corresponding 3D streamlines derived from these slices. Fig 14 quantitatively outlines the results of the three-channel flow curve observed within the original steel cord sample.

The following points can be explicitly established. (1) As the displacement pressure difference decreases, a corresponding decrease in flow velocity is noted. Through variations in displacement differential pressure values, it was observed that at a displacement pressure difference of 3.3273 Pa, the local lattice velocity approached zero. This value 3.3273 Pa represents the critical threshold for displacement differential pressure in the original steel cord sample. Below this threshold, the observed seepage behavior within the original steel cord sample ceases to occur. (2) There are a total of three flow channels identified. Channel A exhibits an average flow velocity of 2.53091×10^−4^ m/s, with the maximum flow velocity within the main channel reaching 4.4852×10^−4^ m/s. The average flow rates for channel B and C were determined as 1.41425×10^−4^ m/s, 2.74879×10^−4^ m/s, respectively, with their respective maximum flow rate measured at 5.6433×10^−4^ m/s and 4.3858×10^−4^ m/s. Notably, the results indicated that the average velocity in channel C surpasses that of the other two channels, while the maximum velocity in channel B exceeds that of the remaining channels. (3) The reduced flow rate at the bottom compared to the top is attributed to increased drag and the presence of smaller disconnected pores within the pore network. (4) As the lattice number X ranges from 0 and 57, a gradual decline in flow velocity occurs, primarily attributed to the progressive narrowing of the channel width. Conversely, within the lattice number X range of 57 and 100, the flow velocity within channels B and C experiences a gradual increase, attributable to the progressive enlargement of the pore channel.

**Fig 15:**
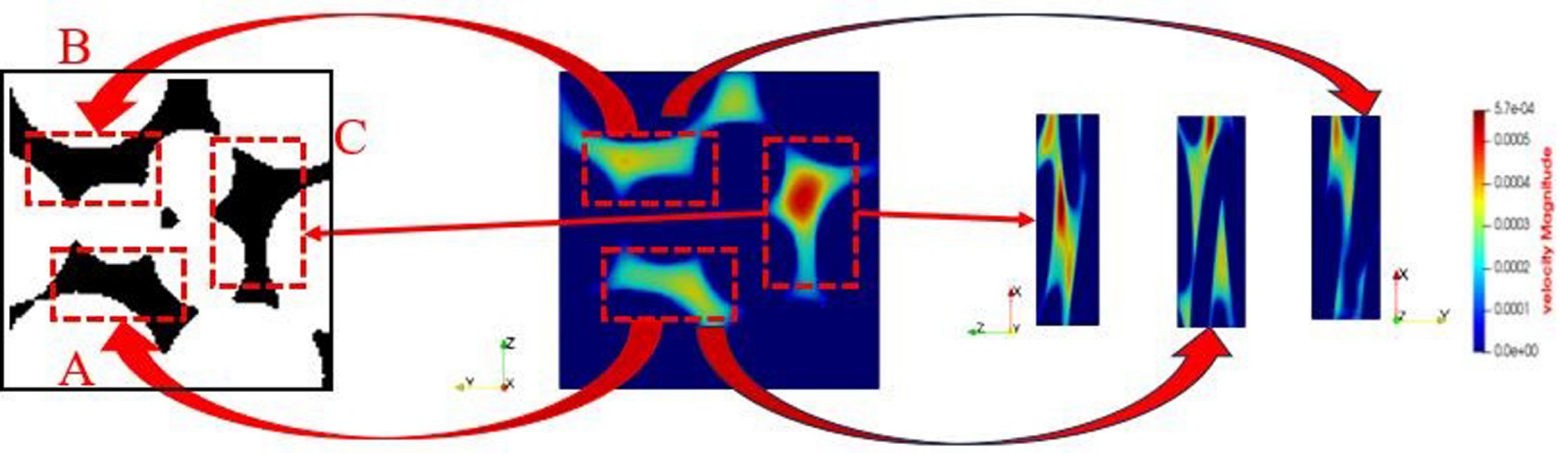
Flow channel structure of tensile sample.

Building upon the aforementioned methodology, a microscopic investigation into the water flow characteristics within the tensile steel cord sample was conducted. This phenomenon exhibited similarities to those observed in the original steel cord sample, indicating that the pore structures retained homogeneity even after undergoing an 800 N stretching force. The channel structure distribution within the tensile steel cord material predominantly consists of three segments: flow channels A, B and C, as depicted in Fig 15.

**Fig 16:**
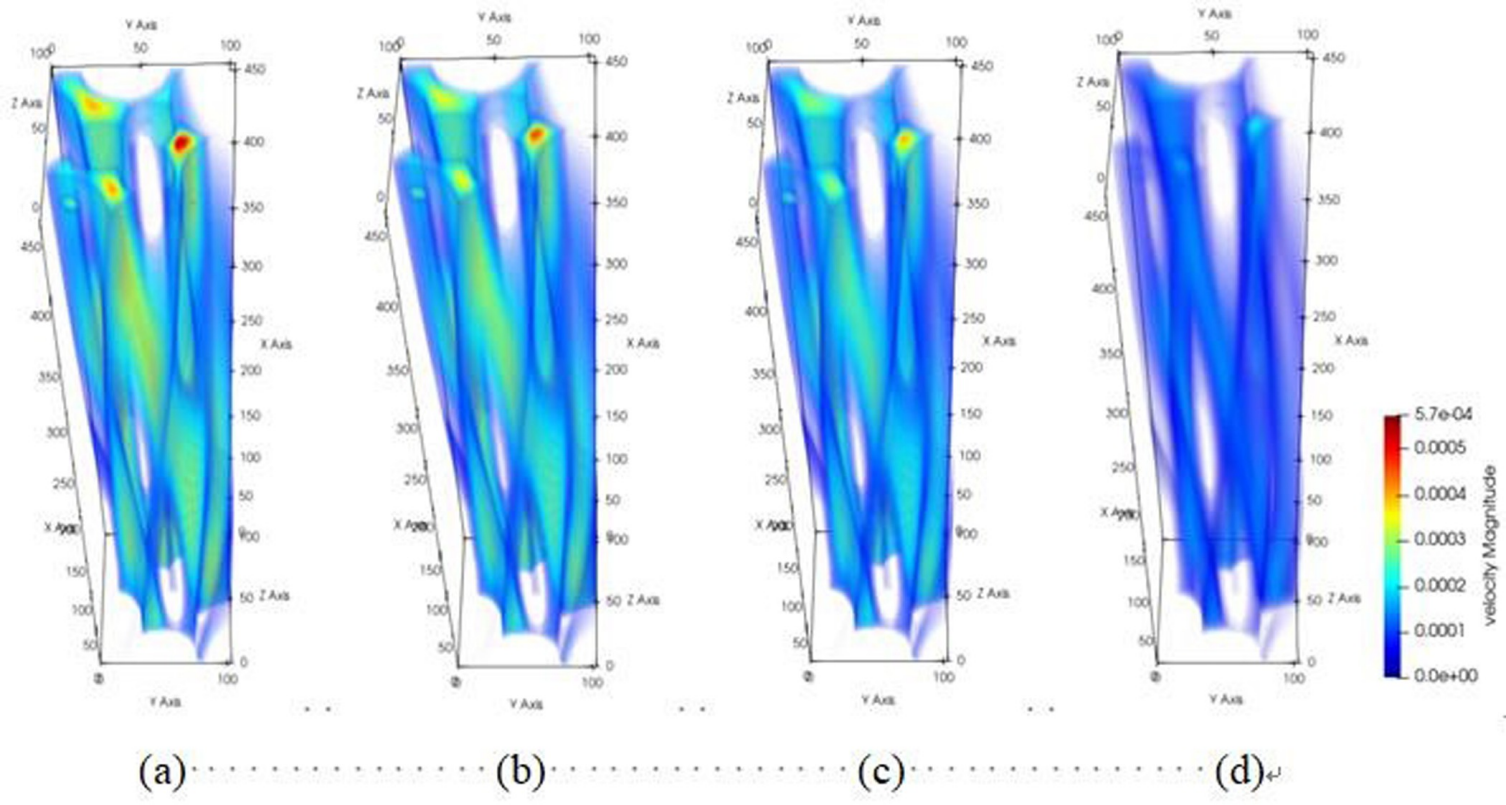
Results of 3D flow field of tensile sample.

**Fig 17:**
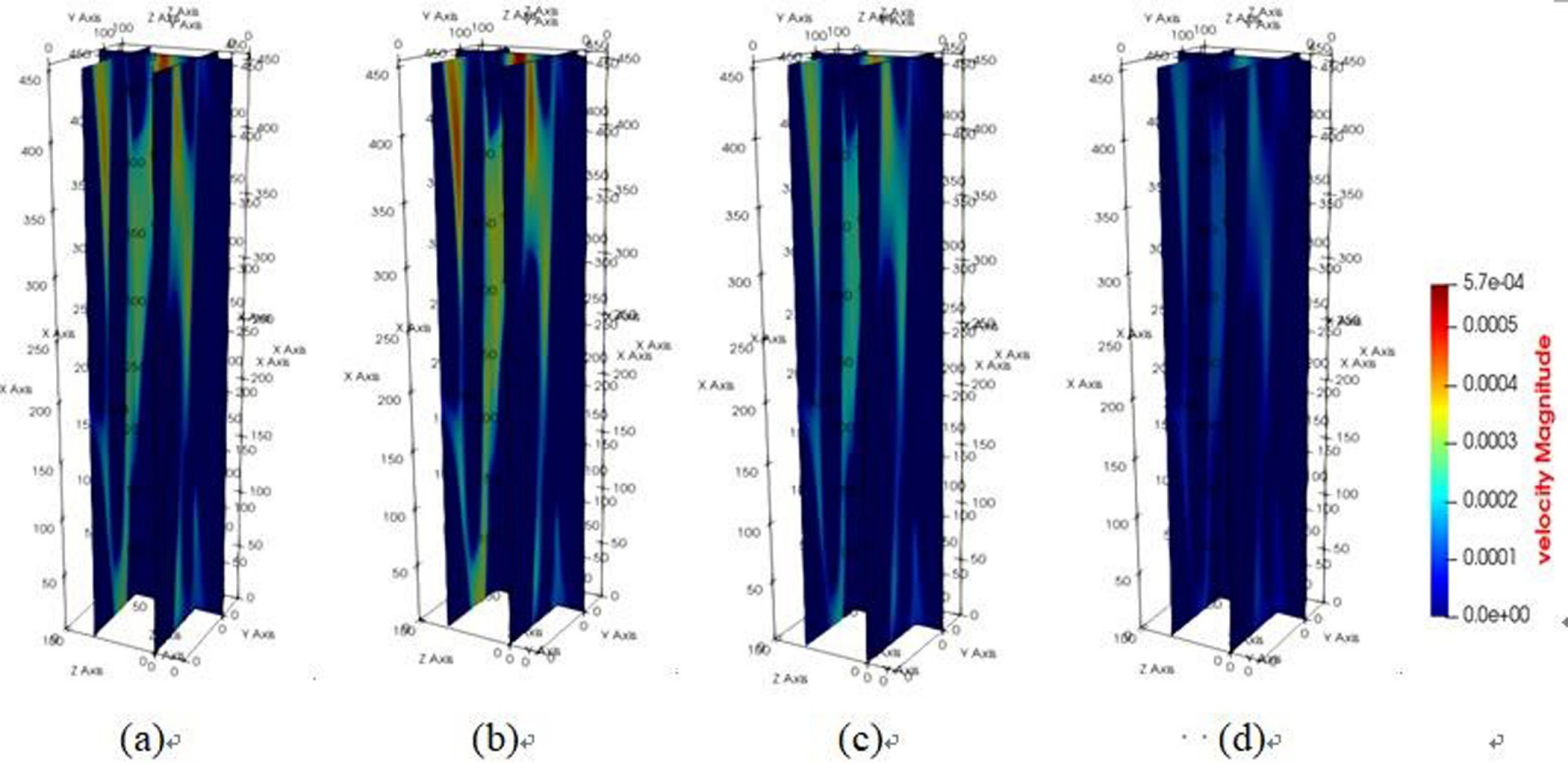
Results of 2D flow slice of tensile sample.

**Fig 18:**
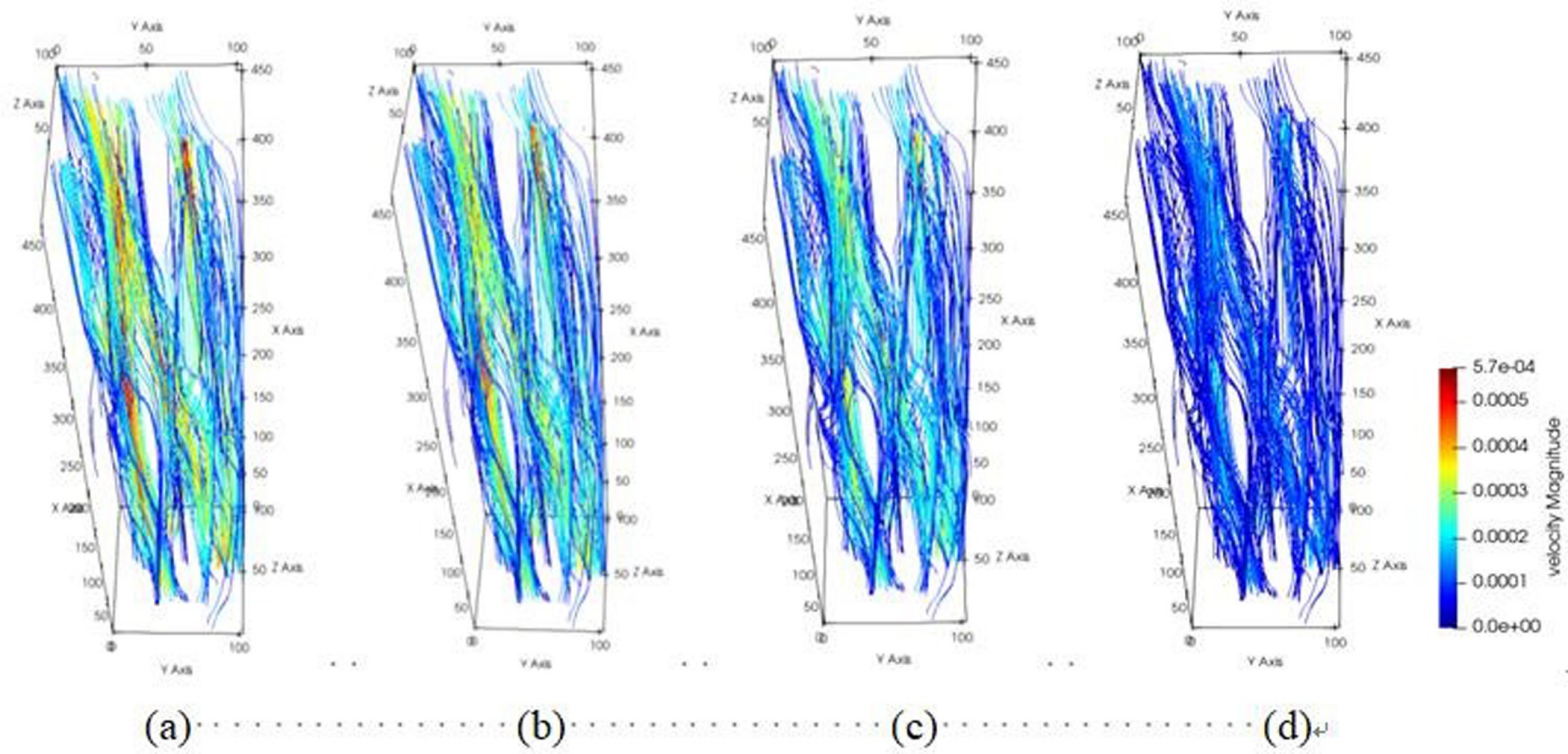
Results of 3D streamline of tensile sample.

**Fig 19:**
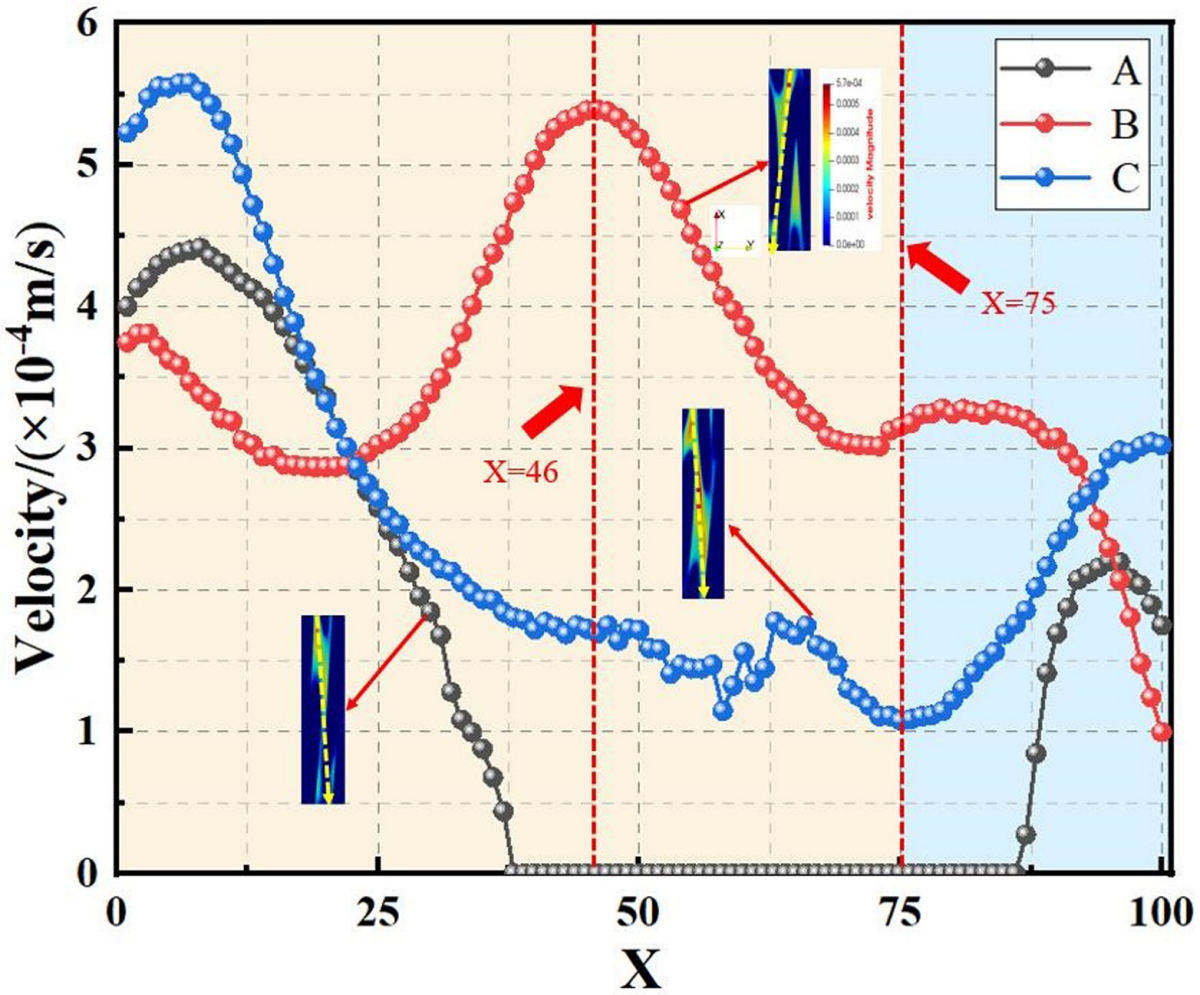
Results of three-channel curve in tensile sample.

The diagrams depicting the 3D flow fields, 2D flow slices, and 3D streamlines of water flow within the tensile steel cord sample under four displacement differential pressures are showcased in Figs 16–18. Additionally, Fig 19 provides quantitative results illustrating the three-channel flow curve observed in the tensile steel cord sample. Explicitly, the following points can be discerned: (1) A decrease in flow pressure difference corresponds to a reduction in flow velocity within the tensile steel cord sample. The critical value of displacement differential pressure for this sample was measured at 2.6122 Pa, which is lower than the critical value determined for the original sample. When the original steel cord was subjected to an 800 N force, it resulted in decreased porosity, an increased pore radius, and widened flow channels. These changes led to a lower critical value of displacement differential pressure in the tensile steel cord sample. (2) There are three identified flow channels in total. Channel A exhibits an average flow velocity of 1.3796×10^−4^ m/s, while the maximum flow velocity within the main channel reaches 4.4164×10^−4^ m/s. The average flow rates for channel B and channel C were measured at 3.54×10^−4^ m/s, 2.47018×10^−4^ m/s, respectively, with their respective maximum flow rates recorded at 5.3888×10^−4^ m/s and 5.5779×10^−4^ m/s. (3) When the lattice number X ranges between 0 and 46, there is a gradual decline in the flow velocity of channels A and C, while channel B experiences a gradual increase. This trend occurs due to the progressive reduction in pore channels for A and C and an increase in pore channels for B. However, between the lattice number X ranges of 46 and 100, channel B undergoes a gradual decrease in flow velocity, attributed to increased flow resistance. Within the lattice number X range of 75 to 100, there is a gradual increase in the flow velocity of channels A and C, resulting in an outlet flow velocity smaller than the inlet flow velocity. This change is due to the gradual widening of the flow channel.

#### 3.1.3 Pore threshold analysis

In general, 3D CT images of the samples were employed for flow numerical simulation analysis, particularly for estimating permeability. Nonetheless, owing to the intricate pore geometry and relatively low image resolution, uncertainties arose during the segmentation process of distinguishing pores from solid structures based on image pixels.

**Fig 20:**
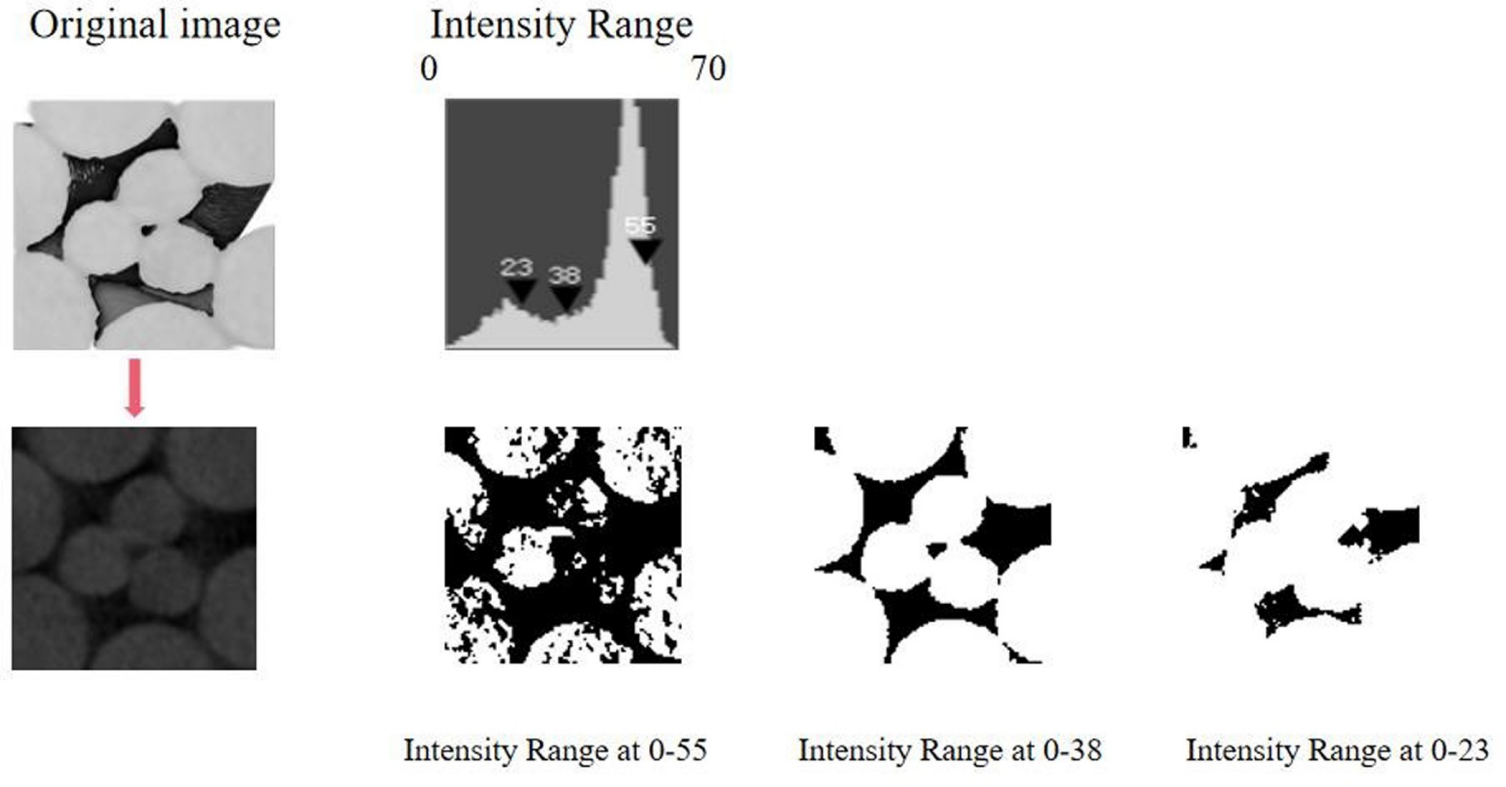
Density range determination in original sample.

During the thresholding process in the Avizo software, the segmentation of pore and solid structures within the original steel cord sample was achieved using the Interactive Threshold module. The grayscale image underwent conversion into a binary format through the binary methodology. Fig 20 illustrates the specific density ranges applied in the image threshold segmentation for pore extraction. Three density ranges of 0–55, 0–38, and 0–23 were selected for this purpose. The clear porous phase, characterized by dark areas surrounded by gray black regions, was designated as the porous phase, while the open bright areas were identified as the solid phase. Observations revealed that when the density ranged from 0 to 55 and 0 to 23, as depicted in Fig 20, certain gray-marked pore areas were not entirely selected, leading to a diminished accuracy in determining the porosity value. Optimal consistency between the pore phase and the segmented region was achieved when employing the density range from 0 to 38. This determination bears direct significance on fluids velocity and flow behavior, as observed in the LBM simulation.

#### 3.1.4 Boundary condition analysis

**Fig 21:**
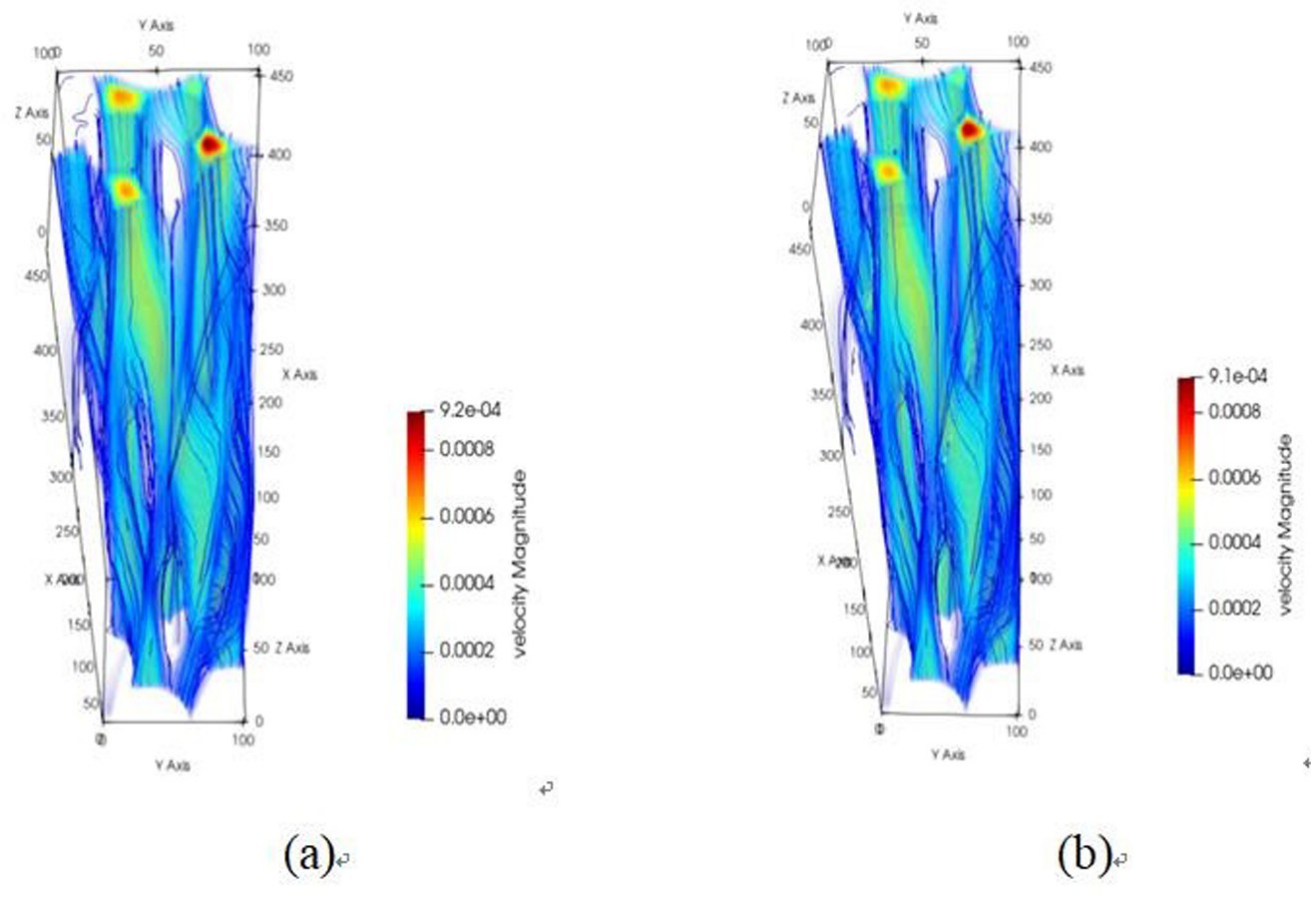
Results of original sample for two boundaries.

**Fig 22:**
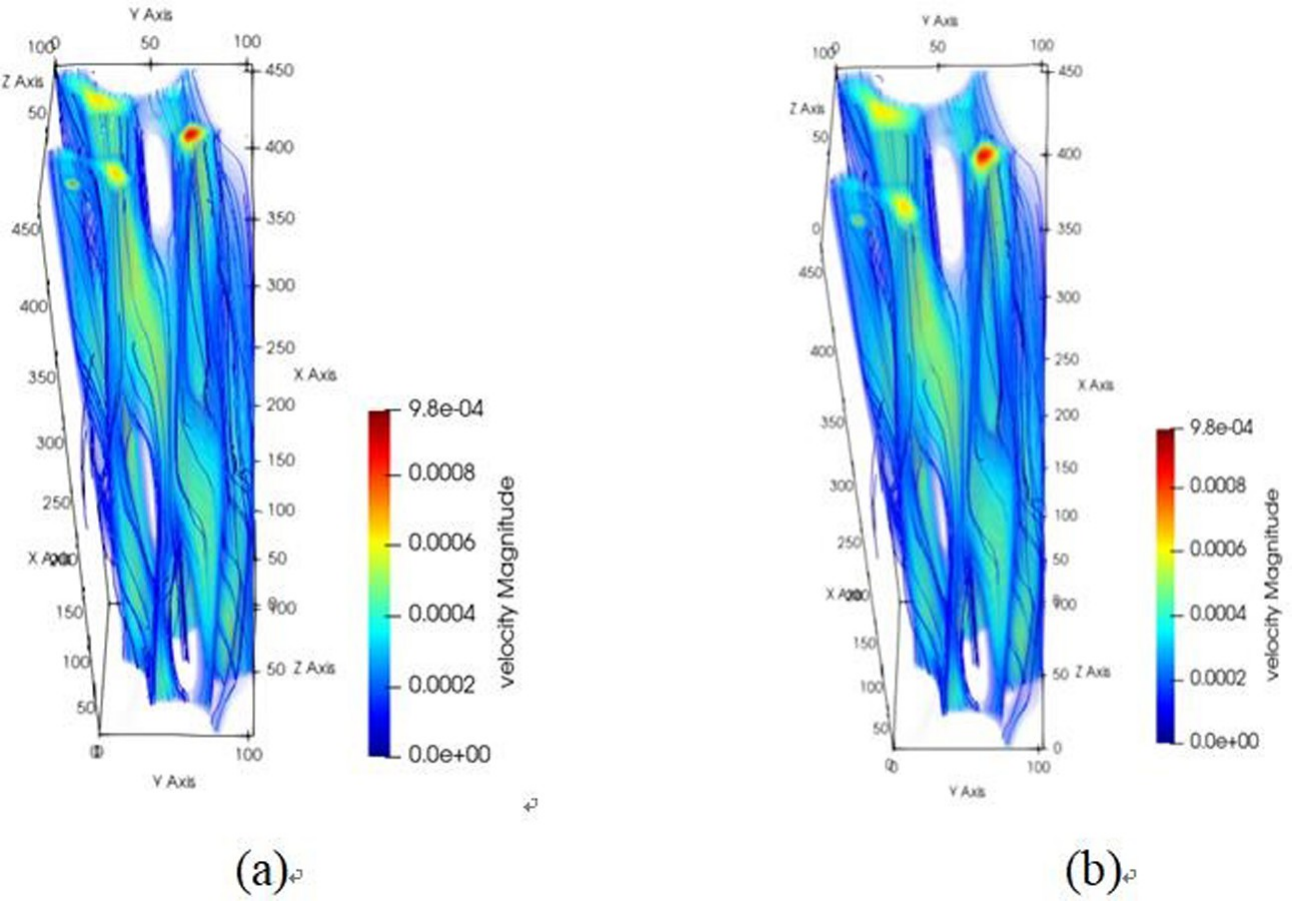
Results of tensile sample for two boundaries.

This section delves into examining the influence of the Zou-He Boundary and the Regularized Boundary on the simulation outcomes concerning fluid flow in both the original and tensile steel cord samples. The simulation parameters were standardized: a displacement differential pressure of 10 Pa, the fluid kinematic viscosity set at 0.0025 m^2^/s, and fluid density established at 1000 kg/m^3^. Figs 21 and 22 present the simulation outcomes for both the original steel cord sample and the tensile steel cord sample, while Tables 3 and 4 explicitly enumerate the average velocity and relative error values observed under the two boundary conditions. The relative error in the calculated average velocities was identified as 1.316 percent and 4.315 percent for the Zou-He Boundary and Regularized Boundary conditions, respectively. These findings elucidate the distinct impacts of these boundary conditions on the accuracy of the simulation results for both original and tensile steel cord samples.

**Table 3:** The average velocities and their relative errors of original steel cord sample under the conditions of Zou-He Boundary and Regularized Boundary.

Boundary conditions	Average velocities (m/s)	Relative errors (%)
Zou-He Boundary	2.051×10^−4^	-
Regularized Boundary	2.024×10^−4^	1.316

**Table 4:** The average velocities and their relative errors of tensile steel cord sample under the conditions of Zou-He Boundary and Regularized Boundary.

Boundary conditions	Average velocities (m/s)	Relative errors (%)
Zou-He Boundary	2.526×10^−4^	-
Regularized Boundary	2.417×10^−4^	4.315

### 3.2 REV-scale LBM results

#### 3.2.1 Construction of pore structures

**Fig 23:**
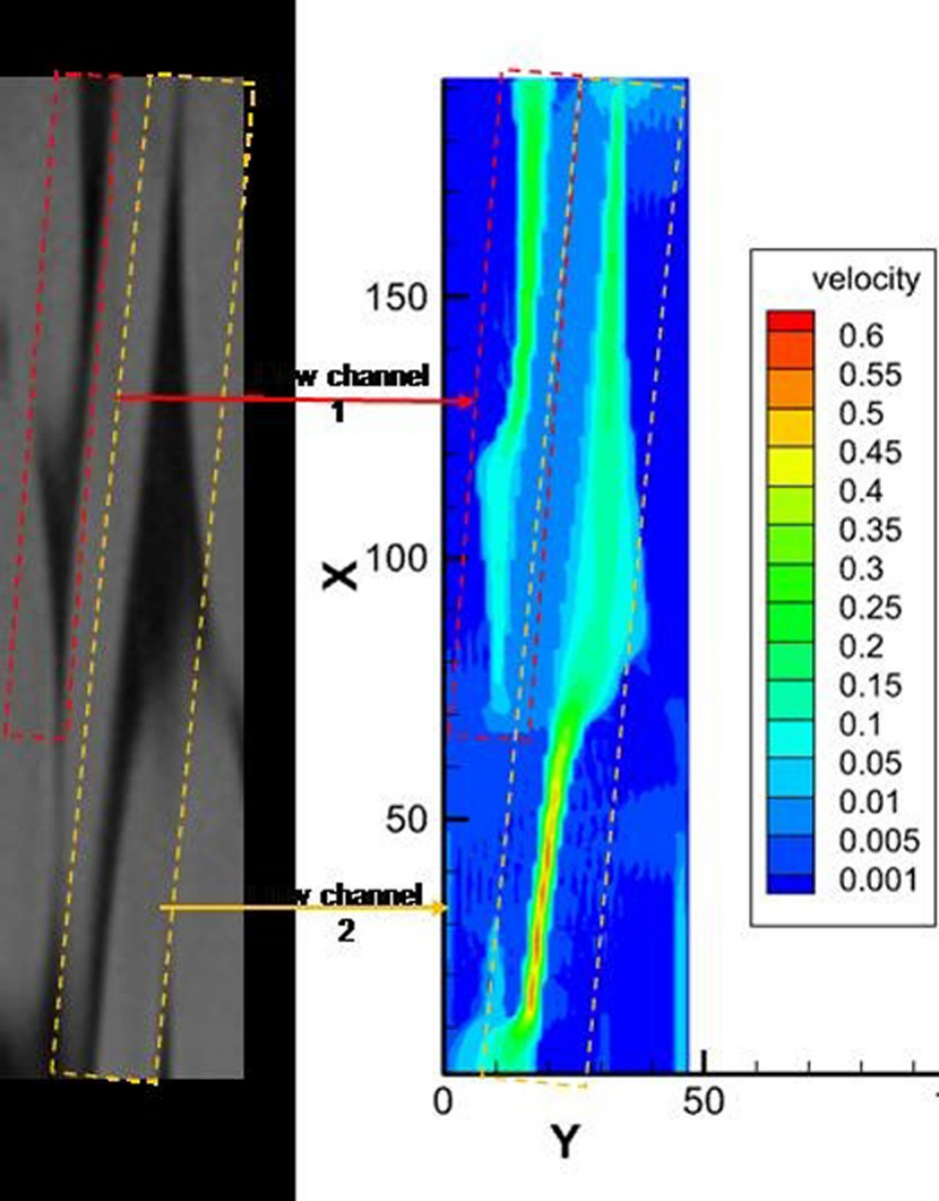
Pore structure and velocity field distribution.

Utilizing CT scan data, the stretched steel cord material underwent a two-dimensional reconstruction process followed by binarization. The material exhibits a spectrum of pores ranging from millimeter-scale to micro-nano dimensions. At the REV scale, meticulous attention to specific seepage behavior details is no longer warranted due to the absence of a solid medium among internal pores within the material. In this context, the focus primary shifts towards altering porosity rather than assigning corresponding pore diameters. For the pores situated between steel cords, the void channels are notably broad, allowing for the derivation of pore diameters based on pore-scale simulation outcomes. The average value for pore radius parameters measures 63.572 μm. Furthermore, the representation of the pore channel between the steel cords can be achieved through a binary image, thereby obviating the necessity to assign its porosity. The reconstructed two-dimensional structure of the steel cord sample spans 47 μm × 192 μm, comprising a discrete lattice consisting of 47 × 192 lattice units. This reconstructed structure is depicted in Fig 23.

#### 3.2.2 Fluid flow characteristics

Fig 23 illustrates the outcomes of LBM simulation depicting water flow within steel cord samples under four different displacement pressure differences. The fluid viscosity is noted as 1.006×10^−6^ m²/s. The visualization in Fig 23 reveals the presence of a primary seepage channel labeled as “Flow channel 2,” and a secondary flow channel denoted as “Flow channel 1” within the steel cord sample. The main seepage channel, recognized by its robust connectivity and substantial width, notably influences the observed flow dynamics.

**Fig 24:**
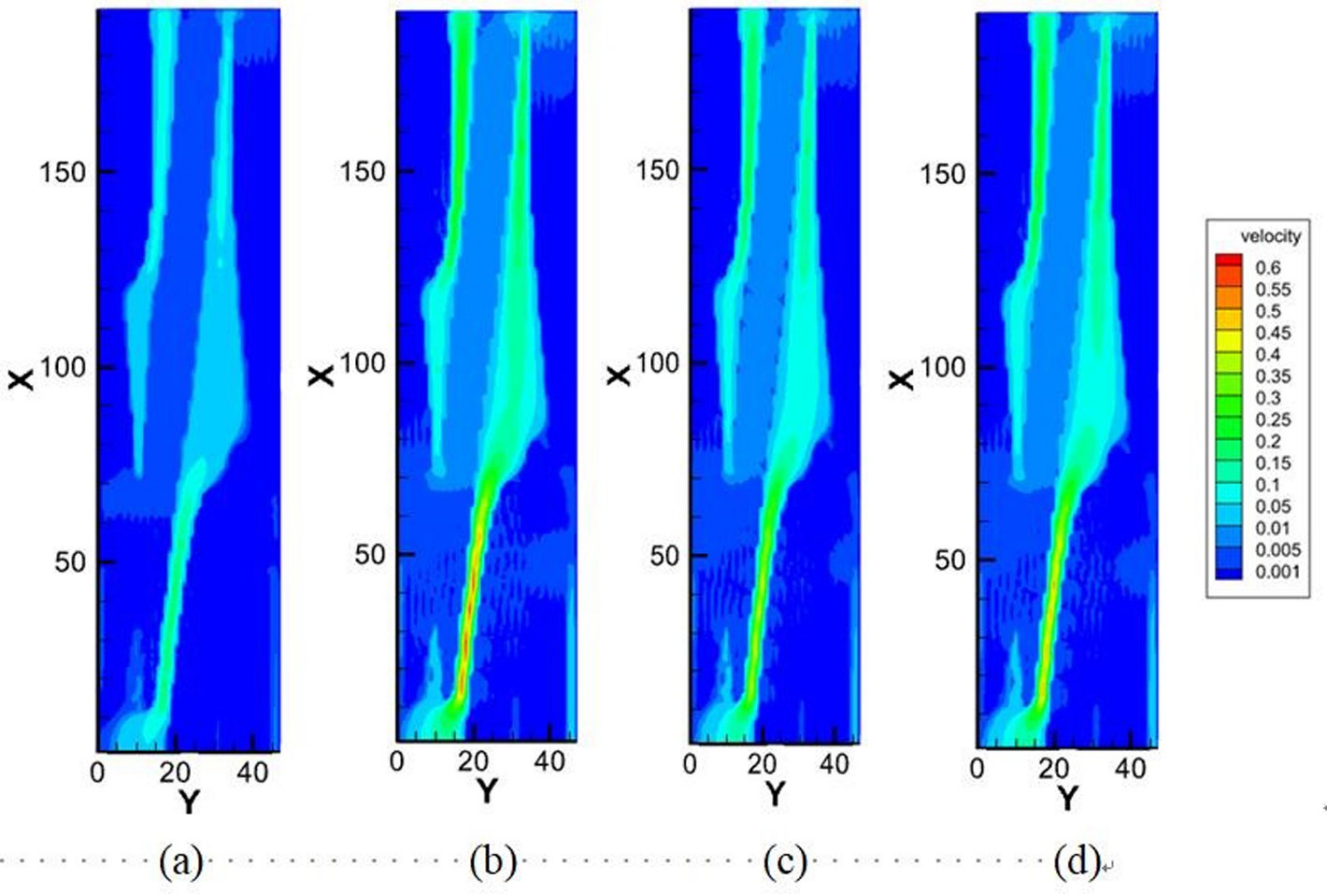
Four pressure differences in tensile sample.

**Fig 25:**
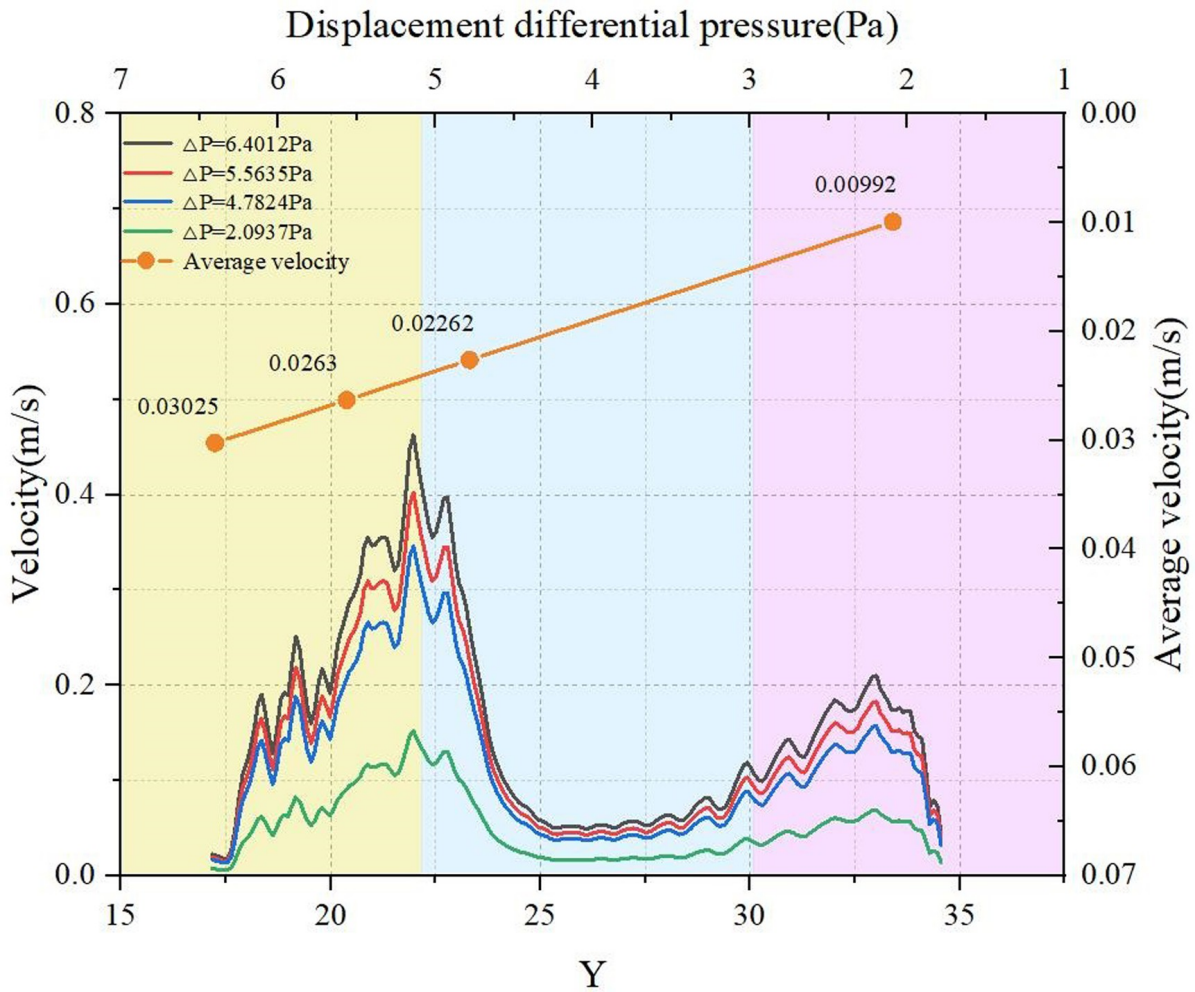
Average velocity of tensile sample.

Figs 24 and 25 describe the outcomes of LBM simulation illustrating the water transmission behavior within steel cord samples under four distinct displacement pressure differences. These can be seen depicted from the figure: (1) As the driving pressure difference steadily decreases, the seepage velocity of water within the steel cord sample progressively diminishes. (2) When the displacement pressure difference is 2.0937 Pa with the lattice units at Y = 14.452 and Y = 26.045, the velocity register at 0.00585 m/s and 0.0163 m/s, respectively, while the fluid velocity within the channel nears zero. Consequently, for the steel cord sample, this signifies a critical threshold at 2.0937 Pa for the displacement pressure difference. When the displacement pressure difference falls below 2.0937 Pa, the steel cord sample ceases to exhibit seepage behavior in the water phase. (3) In the same flow channel of the steel cord samples, a consistent distribution pattern of seepage velocity is observed across the four displacement pressure differences. Within flow channel 2, identified as the primary seepage channel, two distinct peak values and one lower peak value of water seepage velocity are noticeable. Notably, the highest flow rate occurs within the narrow channel, while the lower peak value emerges within the broader channel. The behavior stems from the response observed under the same displacement pressure difference: the fluid tends to disperse more extensively within the wider channel, thereby weakening its force and resulting in a slower flow rate. Conversely, the narrower channel concentrates the flow, enhancing its force and consequently leading to a faster flow rate. (4) Due to the microporous nature of steel cord samples, the percolation of the water phase within these samples requires an initial pressure difference to overcome capillary resistance. Consequently, the initial flow rate of water tends to be low. Under a consistent displacement pressure difference, disparate locations within the sample necessitate varied starting pressure differences, resulting in differing initial velocities. For instance, when △P = 6.4012 Pa, with lattice units X = 200, Y = 17.18, Y = 25.05, and Y = 34.18, the initial velocities of water are 0.02252 m/s, 0.05731 m/s, and 0.11216 m/s, respectively. (5) As the displacement pressure difference escalates, both the maximum and average water transmission velocities within the main seepage channel exhibit an increase, although the amplification rates differ. For instance, considering△P = 2.0937 Pa and △P = 6.4012 Pa as reference points, the maximum transmission velocities of water in the main seepage channel measure 0.130 m/s and 0.397 m/s, respectively. Simultaneously, the corresponding average velocities are 0.00992 m/s and 0.03025 m/s. Evidently, the increase in the displacement pressure difference by 2.067 times corresponds to a 2.053 times increase in maximum velocity and a 2.049 times increase in average velocity within the main seepage channel.

#### 3.2.3 The influence of internal porosity in the steel cord sample

**Fig 26:**
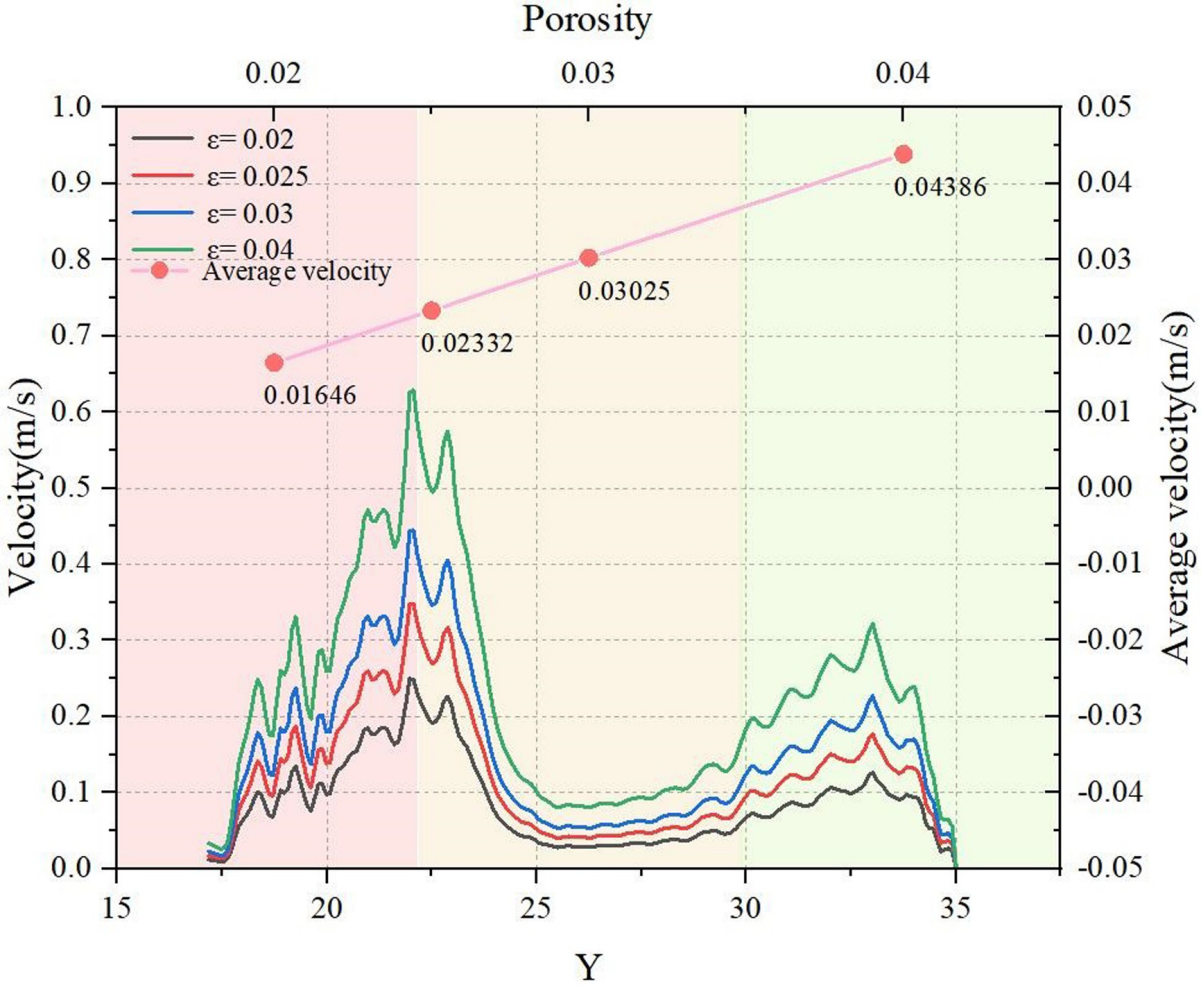
Average velocity in four samples.

In this part, LBM simulation were conducted to replicate water transport within samples of four distinct steel cord bundles. The displacement pressure difference is set at 6.4012 Pa, while the internal porosity (*ε*) of the steel cord bundle varies, specifically 0.02, 0.025, 0.03 and 0.04 for each bundle. Fig 26 delineates the flow velocity distribution and average velocity of water within the main seepage channel of the steel cord samples. The data from Fig 26 demonstrates a notable trend as the increase of the *ε* value, the water flow velocity within the pore intensifies, consequently elevating the velocity distribution within the same channel. Across samples with varying porosities, it’s noticeable that the higher seepage velocity values predominantly occur at the same position within the corresponding pore channel. As the internal porosity of the steel cord increases, both the maximum and average transfer velocities of water within the main seepage channel rise. However, the amplification factor varies. For instance, considering porosity values of *ε* = 0.02 and *ε* = 0.04, the maximum transmission velocities of water in the main seepage channel measure 0.24953 m/s and 0.62882 m/s, respectively. Simultaneously, the corresponding average transmission velocities are 0.01646 m/s and 0.04386 m/s. This indicates that when the internal porosity value of the steel cord is increased by 1 time leads the maximum speed to increase by 1.52 times, and the average speed is increased by 1.66 times.

### 3.3 Comparison of LBM results for the tensile cord sample between pore-scale and REV-scale

Based on the simulated results for the tensile cord sample obtained from both the pore-scale and REV-scale LBM approaches, key observations and conclusions can be drawn: The water seepage velocity increased with the increase of the displacement differential pressure, presenting a good linear trend. This phenomenon should be attributed to the better homogeneity of pore structures in the cord sample, which perfectly accorded with the actual seepage mechanism. Based on the simulation of cord samples by pore-scale and REV-scale LBM, the critical values of displacement differential pressure are different, which are 2.6122 Pa and 2.0937 Pa, respectively. The reasons for this phenomenon can be summarized as follows: (1) Pore-scale LBM has great advantages in simulating microscopic seepage, but is limited to small-size porous media. In contrast, REV-LBM volume averages the statistically averages the porous media’s characteristics, endowing the interior of cord bundles with porosity and enhancing flow capacity. (2) Pore-scale LBM uses 3D data serves as the simulation medium, allowing a clear visualization of velocity distribution near solid walls. However, observing internal flow dynamics within the material proves challenging. To address this, we extracted velocity fields in 2D slices, aligned with the fluid flow direction (i.e., yz direction). Comparing 2D and 3D velocity fields, we noted that the fluid velocity appeared discontinuous in 3D slices while presenting a continuous pattern in 2D diagrams. This disparity emphasized the superior connectivity of pore-throat structures evident in the 2D representation.

## 4. Conclusions

1. The pore structures of the original and tensile steel cord samples were quantitatively analysed, where illustrated that the porosity of the original steel cord sample (20.20%) was higher than that of the tensile steel cord sample (17.69%). However, the average pore radius of the original steel cord sample (39.798 μm) was lower than that of the tensile steel cord sample (63.572 μm).
2. The fluid velocities increased with the increase of displacement differential pressures in the original and tensile steel cord samples, however, presenting the different critical values of displacement differential pressure 3.3273 Pa and 2.6122 Pa, respectively. This phenomenon should be attributed that when the original steel cord sample was loaded with 800 N force, its porosity decreased, its pore radius increased, and then its flow channel became wider, resulting in the lower critical value of displacement differential pressure.
3. The 1/2 sections of 3D construction images in the original and tensile steel cord samples were compared when the displacement differential pressure was determined as 6.4012 Pa, it can be elucidated that the maximal and average seepage velocities at the 1/2 sections in the original steel cord sample were both less than those of tensile steel cord sample. This can be explained that when the original steel cord sample was loaded with 800 N force, it realized the smaller porosity, however, the better connectivity, and accordingly improved the flow velocity.
4. As the internal porosity of the tensile steel cord sample increases, both the maximum and average transfer velocities of water within the main seepage channel rise. However, the amplification factor varies. When the internal porosity value of the steel cord is increased by 1 time leads the maximum speed to increase by 1.52 times, and the average speed is increased by 1.66 times.
5. For the original steel cord sample, the pore phase presented the best consistency with the segmentation area when the density range was determined as 0–38.
6. The effects of Zou-He Boundary and Regularized Boundary on the simulation results of fluid flow in the original and tensile steel cord samples were elucidated. It can be reached that the relative error of calculated average velocities was smaller depending on these two boundary conditions, merely 0.2602%.

## Abbreviations

wi: weight coefficient
ρ: mean fluid density, Kg/m^3^
ei: discrete velocity, m/s
c: lattice velocity
f ^eq^: equilibrium distribution functions
*f*(*x*, *t*): distribution function for the particle with velocity e_i_ at position x
τ: relaxation time
δt,δx: time and length increment (the lattice units)
Cs: sound velocity, m/s
∇P: pressure gradient
μ: dynamic viscosity, Pa‧S
Ω: collision term
v: spherical particle volume
ρ: non-dimensional average fluid density, Kg/m^3^
D: tube diameter
ux: velocity in the x-axis direction, m/s
u: volume averaged fluid velocity (the lattice units)
